# Core Molecular Clock Factors Regulate Osteosarcoma Stem Cell Survival and Behavior via CSC/EMT Pathways and Lipid Droplet Biogenesis

**DOI:** 10.3390/cells14070517

**Published:** 2025-03-31

**Authors:** Sukanya Bhoumik, Yool Lee

**Affiliations:** 1 Department of Translational Medicine and Physiology, Elson S. Floyd College of Medicine, Washington State University, Spokane, WA 99202, USA; sukanya.bhoumik@wsu.edu; 2 Department of Integrative Physiology and Neuroscience, College of Veterinary Medicine, Washington State University, Pullman, WA 99164, USA; 3 Sleep and Performance Research Center, Washington State University, Spokane, WA 99202, USA; 4 Steve Gleason Institute for Neuroscience, Washington State University, Spokane, WA 99202, USA

**Keywords:** circadian clock, cancer stem cell, osteosarcoma, epithelial–mesenchymal transition, lipid droplet

## Abstract

The circadian clock, an intrinsic 24 h cellular timekeeping system, regulates fundamental biological processes, including tumor physiology and metabolism. Cancer stem cells (CSCs), a subpopulation of cancer cells with self-renewal and tumorigenic capacities, are implicated in tumor initiation, recurrence, and metastasis. Despite growing evidence for the circadian clock’s involvement in regulating CSC functions, its precise regulatory mechanisms remain largely unknown. Here, using a human osteosarcoma (OS) model (143B), we have shown that core molecular clock factors are critical for OS stem cell survival and behavior via direct modulation of CSC and lipid metabolic pathways. In single-cell-derived spheroid formation assays, 143B OS cells exhibited robust spheroid-forming capacity under 3D culture conditions. Furthermore, siRNA-mediated depletion of core clock components (i.e., *BMAL1*, *CLOCK*, *CRY1/2*, *PER1/2*)—essential positive and negative elements of the circadian clock feedback loop—significantly reduced spheroid formation in 143B CSCs isolated from in vivo OS xenografts. In contrast, knockdown of the secondary clock-stabilizing factor genes *NR1D1* and *NR1D2* had little effect. We also found that knockdown of *BMAL1, CLOCK*, or *CRY1/2* markedly impaired the migration and invasion capacities of 143B CSCs. At the molecular level, silencing of *BMAL1, CLOCK,* or *CRY1/2* distinctly altered the expression of genes associated with stem cell properties and the epithelial–mesenchymal transition (EMT) in 143B CSCs. In addition, disruption of *BMAL1*, *CLOCK*, or *CRY1/2* expression significantly reduced lipid droplet formation by downregulating the expression of genes involved in lipogenesis (e.g., *DGAT1*, *FASN*, *ACSL4*, *PKM2*, *CHKA*, *SREBP1*), which are closely linked to CSC/EMT processes. Furthermore, transcriptomic analysis of human OS patient samples revealed that compared with other core clock genes, *CRY1* was highly expressed in OS tumors relative to controls, and its expression exhibited strong positive correlations with patient prognosis, survival, and LD biogenesis gene expression. These findings highlight the critical role of the molecular circadian clock in regulating CSC properties and metabolism, underscoring the therapeutic potential of targeting the core clock machinery to enhance OS treatment outcomes.

## 1. Introduction

The circadian clock is a cell-autonomous timing system that generates roughly 24 h periodic rhythms and is conserved in nearly all life, from unicellular organisms to humans [1]. In all living organisms, the fundamental unit of biological timing is the cell [2]. In mammals, including humans, circadian rhythms are primarily governed by a cell-autonomous molecular feedback loop. Within this system, the circadian transcriptional activators BMAL1 (aryl hydrocarbon receptor nuclear translocator-like) and CLOCK (circadian locomotor output cycles kaput) cyclically stimulate the expression of their own repressors, PER (period) and CRY (cryptochrome) [3]. This core oscillatory mechanism is further regulated by an additional complementary loop in which the cycling expression of BMAL1 is controlled by the repressor REV-ERBα/β and the activator RORα/β [3]. Collectively, the molecular clockwork has been reported to regulate a variety of physiological processes, such as differentiation, proliferation, and metabolism, in both normal and cancer cells [4,5,6]. Furthermore, a growing number of studies suggest that circadian clock factors regulate the cell cycle and metabolism associated with malignant cancer progression and anti-tumor drug efficacy [4,7,8,9].

Cancer cells within a tumor exhibit significant heterogeneity, displaying diverse phenotypic and functional characteristics within the complex tumor microenvironment [10]. At the apex of this hierarchical organization resides cancer stem cells (CSCs), a distinct subpopulation characterized by self-renewal capacity and multipotency [11]. CSCs are widely recognized as the primary drivers of tumor initiation, recurrence, metastasis, chemoresistance, and, ultimately, mortality [12]. Phenotypically, CSCs are frequently associated with the epithelial–mesenchymal transition (EMT), a cellular program that endows cancer cells with enhanced motility and invasiveness, which are hallmarks of malignant stem-like cells [13,14,15,16,17]. Recent advancements in stem cell biology and associated technologies have facilitated the identification and characterization of CSC/EMT populations in patient-derived samples and in vitro models, such as tumor spheroids, providing valuable insights into the underlying mechanisms driving tumor progression and treatment resistance [18,19,20].

Osteosarcoma (OS) is a malignant mesenchymal tumor in which the cancer cells produce osteoid, the organic extracellular matrix of bone [21]. Despite advances in diagnosis and treatment, the prognosis of OS is still poor, and the long-term cure rate for patients has reached a plateau over the last 30 years [22]. Currently, the primary treatment for OS is a combination of surgery, chemotherapy, and radiation therapy. However, OS frequently develops resistance to conventional chemotherapies, resulting in tumor recurrence, mainly due to OS cancer stem cells (OSCs), which share the characteristics of self-renewal, multipotency, tumorigenicity, multiple drug resistance, and metastasis [23,24,25]. Thus, a better understanding of tumor pathology in OS and the mechanisms of initiation and recurrence is urgently needed to improve patient treatment and prognosis. Emerging studies across various cancer types, including glioblastoma and leukemia, have revealed that circadian molecular functions are critical for the survival and maintenance of CSC populations [26,27,28,29]. However, the precise roles and mechanisms of the circadian clock in regulating CSC properties and functionality in OS remain unclear.

Increasing evidence underscores the interplay between metabolic pathways—such as glycolysis, mitochondrial oxidative phosphorylation (OXPHOS), and lipid metabolism—in the regulation of the tumorigenic and invasive properties of CSCs [30,31,32,33,34]. Lipid droplets (LDs), once thought to primarily store lipids, such as cholesterol esters and triacylglycerols (TAGs), are now recognized as key regulators of lipid metabolism and signaling pathways, with their increased presence in CSCs enhancing tumorigenicity and invasiveness in various cancers [35,36,37,38,39,40]. Despite growing evidence of the circadian regulation of lipid metabolism and LD content [41,42,43,44], how the molecular clock modulates LD-linked metabolic pathways in terms of CSC phenotypes has not been fully investigated.

In this study, using a human OS model, we have demonstrated that core clock factors play pivotal roles in regulating OS stem cell survival and behavior by modulating CSC/EMT pathways and LD biogenesis. Depleting components of both the positive and negative limbs of the core clock machinery (e.g., *BMAL1*, *CLOCK*, *CRY1/2*) significantly impaired the spheroid formation, migration, and invasion capacities of 143B CSCs, but depleting secondary clock factors (*NR1D1/2*) had minimal impact. In addition, knockdown of *BMAL1, CLOCK,* or *CRY1/2* altered gene expression profiles associated with stemness and EMT while also reducing intracellular LD formation and the expression of LD biogenesis-related genes. Furthermore, integrated transcriptomic analysis revealed that the expression of *CRY1*, among other core clock components, is high in OS tumors compared to control tissues and is positively correlated with patient survival and LD biogenesis. Taken together, these findings reveal the novel mechanisms underlying the roles of the circadian clock in OS stem cell physiology and provide compelling evidence for the therapeutic potential of targeting the core circadian clock machinery in OS treatment.

## 2. Materials and Methods

### 2.1. Human Osteosarcoma Cell Lines and Culture

The human osteosarcoma cell lines U-2OS, 143B, Saos-2, and MNNG were procured from the American Type Culture Collection (ATCC) and cultured in Dulbecco’s Modified Eagle Medium (DMEM; Gibco, Life Technologies, NY, USA). The medium was supplemented with 10% fetal bovine serum (FBS; Sigma-Aldrich, St. Louis, MO, USA) and 1% penicillin–streptomycin (Gibco, Life Technologies). Cultured OS cells were maintained at 37 °C in a humidified incubator with 5% CO_2_ to ensure optimal growth conditions. For gene silencing studies, Lipofectamine RNAiMAX (Thermo Fisher Scientific, Waltham, MA, USA) was used according to the manufacturer’s protocols to transfect the cells with siRNA.

### 2.2. Transfection of siRNA

To knock down human *CLOCK* (SI00069790), *BMAL1* (GS406), *CRY1* (SI02757370), *CRY2* (GS1408), *PER1* (SI03076752), *PER2* (SI03109687), *NR1D1* (SI00130935), and *NR1D2* (SI03051965), specific siRNAs were purchased from Qiagen. Transfections of U-2OS cells were performed using 100 pmol of siRNA per well and Lipofectamine RNAiMAX Transfection Reagent (13778075, Thermo Fisher Scientific, Waltham, MA, USA), following the manufacturer’s protocol.

### 2.3. Isolation of Cancer Stem Cells from Human OS Xenografted Mice

All animal procedures were carried out in accordance with the National Institutes of Health Guide for the Care and Use of Laboratory Animals and approved by the WSU Institutional Animal Care and Use Committee (IACUC; ASAF# 6895). To isolate stem cells from orthotopically induced human osteosarcoma tumor xenografts, the CD133 MicroBead Kit (130-100-857, Miltenyi Biotech, Bergisch Gladbach, Germany) and the MiniMACS™ Kit (130-090-312, Miltenyi Biotech) were utilized, according to the manufacturer’s instructions. Briefly, for each sample, solid OS tumor tissue was harvested from a xenograft generated by the intratibial injection of 143B cells into 6∼8-week-old BALB/c nude immunodeficient mice (CAnN.Cg-Foxn1nu/Crl, Charles River, Wilmington, MA, USA), as described in our previous study [33]. The tissue was mechanically dissociated into a single-cell suspension using a sterile scalpel, followed by enzymatic digestion with 200 U/mL Collagenase type IV (17104019, Thermo Fisher Scientific, Waltham, MA, USA) and 0.6 U/mL Dispase (D4818, Sigma, St. Louis, MO, USA) to facilitate cell release. The resulting cell suspension was then filtered through a 70 µm cell strainer to remove debris and clumps. Cells were then washed with PBS and resuspended in CD133 MicroBead buffer before being incubated with CD133 MicroBeads for 15–30 min to selectively label CD133-positive stem cells. Labeled cells were then separated using the MiniMACS™ Separator. This involved applying the cell suspension to a mini-column placed in the magnetic field of the separator, resulting in CD133-positive cells being retained on the column and unlabeled cells being washed away. Finally, the CD133-positive stem cells were eluted from the column and used for downstream analyses, such as immunofluorescence staining with anti-CD133 antibodies or 3D spheroid formation, as depicted in the figures.

### 2.4. 3D Cell Culture of Human OS Spheroids

To establish 3D tumor spheroid cultures, osteosarcoma cell lines (U-2OS, 143B, Saos-2, and MNNG) were seeded at a density of 10,000 cells per well in ultra-low-attachment 6-well plates (3471, Corning, NY, NY, USA). The cells were cultured in 3D Tumorsphere Medium XF (PromoCell GmbH, Heidelberg, Germany), a specialized serum-free and xeno-free medium optimized for cancer stem cell (CSC) enrichment and long-term 3D culture. To prevent excessive aggregation, the culture medium was supplemented with 0.5% methylcellulose. The spheroid cultures were maintained in a humidified incubator at 37 °C with 5% CO_2_ for one week, after which spheroid formation was assessed via live-cell imaging using the ZOE Fluorescent Cell Imager (1450031, BioRad, Irvine, CA, USA). For siRNA-based experiments, 143B CSCs were initially seeded at a density of 50,000 cells per well in a 24-well plate (353504, Corning, New York, NY, USA). After 24 h, the cells were transfected with siRNA for 48 h according to the experimental design. Following transfection, the culture medium was collected, and adherent cells were detached using 0.25% trypsin-EDTA. The detached cells were combined with the collected medium, centrifuged, and resuspended in fresh culture medium before being reseeded at 10,000 cells per well in ultra-low-attachment 6-well plates. Tumor spheroids were then cultured for an additional seven days, and their morphology was analyzed using fluorescence imaging.

### 2.5. Immunofluorescence Analysis

For immunofluorescence (IF) analysis, 143B CSCs and non-CSCs isolated from in vivo OS xenografts, as described above, were plated in a 24-well or 98-well plate. The next day, the cells were fixed with 4% paraformaldehyde (PFA, P6148, Sigma-Aldrich, St. Louis, MO, USA) in PBS and incubated with an anti-CD133 antibody (66666-1-Ig, Proteintech, Rosemont, IL, USA) or anti-Ki-67 antibody (9129, cell signaling, Danvers, MA, USA). Following subsequent incubation with a secondary antibody conjugated to Alexa Fluor 568 (A20003, Thermo Fisher Scientific, Waltham, MA, USA) and Hoechst nucleic acid staining dye (62249, Thermo Fisher Scientific, Waltham, MA, USA), the cells were visualized using a Cytation 5 imaging system.

### 2.6. Bioluminescence Recording and Data Analysis

A total of 1.5 × 10^5^ *pPer2* reporter 143B CSCs and non-CSCs were seeded onto each 35 mm dish. After exposing the cells to chronic 100 nM dexamethasone (dex; D2915, Sigma, St. Louis, St. Louis, MO, USA), they were treated with dex, and real-time bioluminescence of the control and jet-lag cells was monitored using a LumiCycle luminometer (Actimetrics, Wilmette, IL, USA), as previously described [21]. The period and amplitude of the luminescence data were determined using the LumiCycle Data Analysis software program (Actimetrics, Wilmette, IL, USA).

### 2.7. Assessment of Apoptosis and Necrosis

Apoptotic and necrotic cell death was evaluated using the Annexin V-Cy3 Apoptosis Staining/Detection Kit with SYTOX (ab14144, Abcam, Cambridge, MA, USA) according to the manufacturer’s one-step staining protocol. Cells were seeded at a density of 2 × 10⁴ cells per well in 96-well tissue culture-treated plates with complete culture medium. After a 24 h incubation period, cells were transfected with either control or clock gene siRNA for 48 h. Following transfection, the medium was removed, and cells were incubated with the fluorescent dyes for 30 min. Labeled cells were then analyzed using a BioTek Cytation 5 Cell Imaging Multimode Reader (Agilent Technologies, Santa Clara, CA, USA), employing the FITC filter channel (Ex. 488 nm/Em. 530 nm) for SYTOX green dye and the rhodamine filter channel (Ex. 543 nm/Em. 570 nm) for Annexin V-Cy3.

### 2.8. In Vitro Cell Migration and Invasion Assays

For the migration assays, 143B CSCs were seeded at a density of 50,000 cells per well of a 96-well plate and transfected with siRNA. After 48 h, a scratch was created in the cell monolayer using a 20 µL pipette tip. Live-cell imaging was conducted over the following 48 h, with representative images captured at 0, 12, and 24 h. Wound closure was quantified using ImageJ (1.53C) software, as described in previous studies [45,46]. For the invasion assays, siRNA-transfected cells were seeded 48 h post-transfection onto 24-well plates fitted with transwell inserts (353097, Corning, NY, USA). To assess the effect of oleic acid (OA) (90260, Cayman Chemical, Ann Arbor, MI, USA) treatment, siRNA-treated cells were exposed to 60 µM OA in DMEM, while control wells received 150 µM BSA-containing DMEM, followed by incubation at 37 °C and 5% CO_2_ for an additional 24 h, as described in [47], before seeding the cells into transwell inserts. After invasion, cells on the underside of the inserts were stained with 0.5% crystal violet solution (405831000, Thermo Fisher Scientific). Brightfield images were acquired using a Cytation 5 imaging system, and the number of invading cells per field was quantified using ImageJ, following previously published protocols [45,46].

### 2.9. Lipid Droplet Detection Analysis

To analyze lipid droplets (LDs), 143B CSCs were plated at 15,000 cells per well on a high-content imaging 96-well plate (4517, Corning, NY, USA) and transfected with control siRNA (si-CTL) or core clock gene targeting siRNA (*si-BMAL1*, *si-CLOCK, si-CRY1*, *si-CRY2*, *si-CRY1/2*), as specified in the figures. After 48 h, the cells were fixed with 4% PFA (P6148, Sigma-Aldrich, St. Louis, MO, USA) and stained with Nile Red (1 μg/mL, 30787, Cayman Chemical Company, Ann Arbor, MI, USA) to visualize LDs. Nuclei were counterstained with Hoechst. Representative images were captured using the red fluorescence filter cube (Ex 531 nm/Em 593 nm) for LDs and the DAPI filter cube (Ex. 377 nm/Em 447 nm) for nuclei on a Cytation 5 multi-mode reader. The number of LDs per cell was quantified from the images using ImageJ (1.53C) software.

### 2.10. RNA Extraction, Reverse Transcription, and Quantitative PCR

Total RNA was extracted from 143B CSCs using the RNeasy Plus Mini Kit (74134, Qiagen, Germantown, MD, USA), following the manufacturer’s instructions. Complementary DNA (cDNA) was synthesized from equal amounts of RNA using the Invitrogen Superscript II RT First-Strand Synthesis System, with 2 μL of random hexamers and 9 μL of RNA, as per the manufacturer’s protocol (Life Technologies, Carlsbad, CA, USA). Quantitative PCR (qPCR) was carried out using SYBR Green PCR Master Mix (1708880, BioRad, Hercules, CA, USA) and 10 μM of forward and reverse primers (Appendix Table A1, Table A2 and Table A3). Reactions were cycled as follows: 50 °C for 2 min, 95 °C for 10 min, followed by 40 cycles of 95 °C for 15 s and 60 °C for 1 min. Reaction specificity was confirmed by melting curve analysis. Gene expression levels were quantified using the comparative Ct method, with normalization to GAPDH as an internal control. All experiments were performed on a ViiA7 Real-Time PCR machine (Thermo Fisher Scientific, Waltham, MA, USA).

### 2.11. Analysis of Differential Gene Expression and Prognostic Correlations in Human OS Patients

To assess the differential expression of the circadian genes *BMAL1*, *CLOCK, CRY1*, and *CRY2* in osteosarcoma (OS) tissues, publicly available RNA sequencing data (GSE99671) from 18 OS patients [48] were analyzed. The dataset consisted of total RNA extracted from 36 fresh–frozen bone tissue samples, including 18 tumoral and 18 paired non-tumoral samples. Fragments Per Kilobase of transcript per Million mapped reads (FPKM) values were calculated for each gene to determine expression differences. Additional differential gene expression in tumor versus normal tissues from human sarcoma (SARC) patients was analyzed using Gene Expression Profiling Interactive Analysis (GEPIA; http://gepia.cancer-pku.cn/; accessed on 1 June 2024), a web-based platform that provides differential gene expression (DGE) analysis, survival analysis, and correlation analysis across various cancer types using data from The Cancer Genome Atlas (TCGA) and Genotype-Tissue Expression (GTEx) projects. Box plots were generated to illustrate the differential expression patterns of core clock genes (*BMAL1*, *CLOCK*, *CRY1*, *CRY2*) in tumor and normal samples. Survival analyses were conducted for *BMAL1*, *CLOCK*, *CRY1*, and *CRY2* using GEPIA. Kaplan–Meier survival curves were generated, and Cox proportional hazard models were applied to evaluate the impacts of gene expression levels on patient outcomes. Finally, correlations between the expression levels of *BMAL1*, *CLOCK*, *CRY1*, *CRY2*, and genes associated with lipid droplet biogenesis were analyzed in human OS patient samples using the GEPIA platform to explore potential links between clock gene regulation and lipid metabolism in OS.

### 2.12. Statistical Analyses

Statistical analyses for all experiments were conducted using GraphPad Software (version 10). Fluorescence intensities, immunoblots, and spheroid numbers and sizes were quantified using ImageJ software, and statistical evaluation of the data was performed using GraphPad. Significance was assessed using two-way ANOVA, one-way ANOVA, or unpaired Student’s *t*-tests, as appropriate. When *p* was less than 0.05, it was considered statistically significant.

## 3. Results

### 3.1. Establishing and Characterizing a CSC Model for Human OS Using 143B Cells

**Figure 1:**
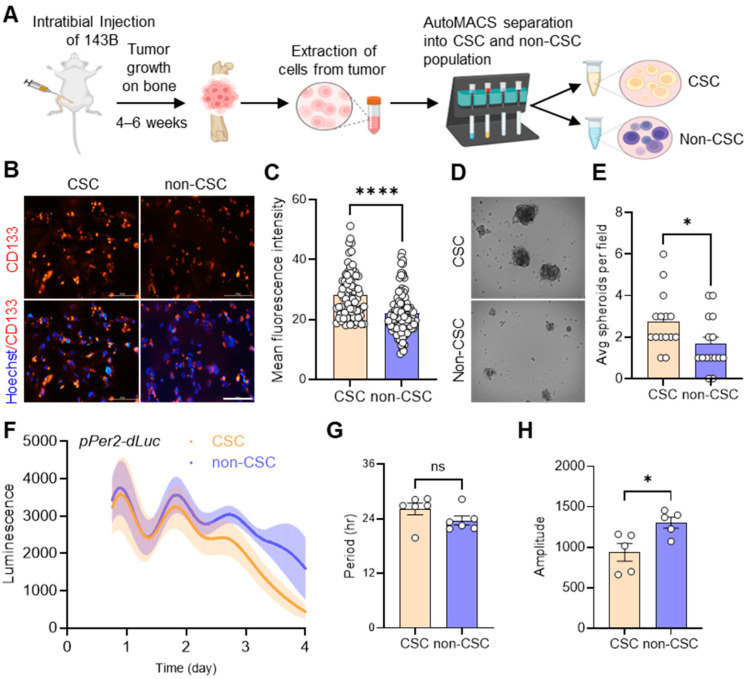
Extraction and functional validation of cancer stem cells (CSCs) from in vivo 143B-driven osteosarcoma. (**A**) 143B OS cells stably expressing *pPer2-dLuc* were injected into mice intratibially. When tumors developed in mice after 4–6 weeks, as confirmed by bioluminescence imaging, they were harvested, dissociated into single cells, and separated into CSC and non-CSC populations using automated magnetic-activated cell sorting (autoMACS). The diagram was created using BioRender (https://www.biorender.com/). (**B**) Representative images showing immunofluorescent staining of endogenous CD133 expression in 143B CSCs and non-CSCs using an anti-CD133 antibody (red). DAPI (blue) merged images are presented in each of the image panels. Scale bar: 10 μm. (**C**) The mean fluorescence intensities of CD133 staining in CSCs and non-CSCs, as shown in B, were determined using ImageJ. (**D**) CSCs and non-CSCs were seeded at a density of 10,000 cells/well on a low-attachment 6-well plate under 0.5% methylcellulose conditions to facilitate spheroid formation. Representative brightfield images of spheroids formed by CSCs and non-CSCs after 1 week in culture are shown. (**E**) The average number of spheroids per field (*n* > 15) for each cell type, as shown in D, was calculated using Image J. (**F**) Real-time bioluminescence recordings of a *Per2* promoter-luciferase reporter (*pPer2*-*dsLuc*) in CSCs (*n* = 5) and non-CSCs (*n* = 5), displaying the mean rhythmic curves (solid lines) with shaded areas representing the error bar ranges. (**G**,**H**) The bar graphs present the period (**G**) and amplitude (**H**) analysis results of (**F**) using Lumicycle Analysis software. All statistical analyses were performed using ImageJ and GraphPad Prism software. * *p* < 0.05 and **** *p* < 0.0001 by Welch’s *t*-test. ns: not significant. All data shown are representative of at least two to three independent experiments.

To establish a CSC research model for human OS, we assessed the feasibility of using a 3D spheroid culture system, which mimics the native tumor environment and supports CSC propagation [49,50,51], with various human OS cell models (U-2OS, 143B, MNNG, Saos-2). Interestingly, our results showed that among the cell lines tested, 143B cells had the highest tumor spheroid formation capacity under 3D stem cell culture conditions (Figure S1). This is consistent with previous studies that characterized 143B cells as a representative model for studying human OS disease due to their enhanced tumorigenic potential and metastasis in mouse xenotransplantation studies in vivo [52,53]. In a recent study, we established a 143B bioluminescent reporter cell line that stably expresses a *Per2* promoter-driven luciferase construct (*pPer2-dLuc*) and verified that these cells form in vivo OS tumors 4–6 weeks after orthotopic xenotransplantation [33]. To isolate CSCs from in vivo OS tumors, we intratibially injected 143B reporter OS cells into the mice. When OS tumors became detectable, they were harvested, dissociated into single-cell suspensions, and separated into CSC and non-CSC populations using the automated magnetic-activated cell sorting (autoMACS) system, which utilizes magnetic beads to isolate CSCs based on the expression of the CSC surface marker CD133 (Figure 1A) [54,55].

To validate our separation of the CSC and non-CSC populations in our tumor samples, we performed immunofluorescence (IF) analysis by staining for CD133 using a specific anti-CD133 antibody. We observed significantly brighter CD133 staining in CSCs compared to non-CSCs (Figure 1B,C). In addition, examining spheroid formation in 3D tumor cell cultures revealed that CSCs exhibited significantly higher rates of spheroid formation and generated larger spheroids compared to non-CSCs (Figure 1D,E). To assess the differential circadian profiles of CSC and non-CSC populations, we performed real-time bioluminescence recording analysis. Our results showed that both CSCs and non-CSCs exhibited distinct circadian oscillations of *Per2* promoter activity, with similar periods (Figure 1F,G). However, the non-CSCs had a moderately higher amplitude of *pPer2-dLuc* expression than the CSCs (Figure 1H). These data suggest that circadian rhythms are present in 143B CSCs isolated from in vivo xenograft OS tumors.

### 3.2. Knockdown of Core Clock Genes Impairs 143B CSC Spheroid Formation

**Figure 2:**
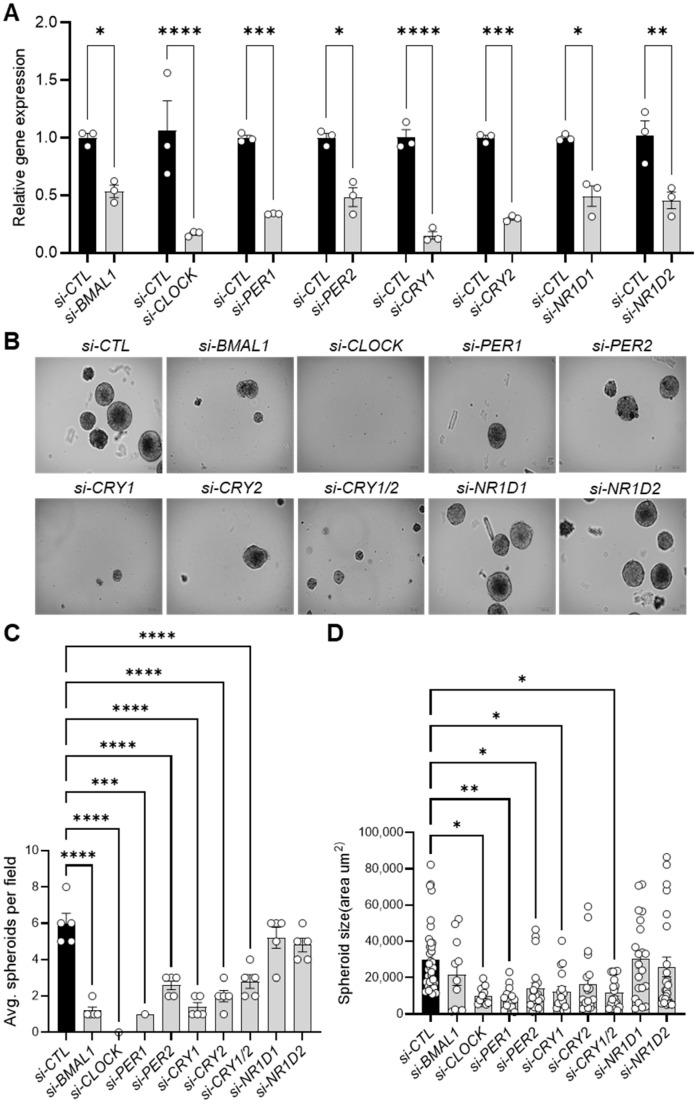
Knockdown of core clock genes reduces spheroid formation in 143B CSCs. (**A**) qPCR analysis validating the RNAi-mediated knockdown of core clock gene expression in 143B CSCs, as indicated, 48 h after the transfection of specific siRNAs. (**B**) Knockdown of core clock genes reduces spheroid formation. 143B CSCs were transfected with the indicated siRNAs for 48 h and then seeded for spheroid formation at a density of 10,000 cells per well in low-attachment 6-well plates. After one week in culture, brightfield images (n > 5) of the siRNA-treated spheroids were captured. Representative images are shown. (**C**,**D**) The numbers (**C**) and sizes (**D**) of the spheroids in (**B**) were quantified using ImageJ software. Statistical significance was determined by one-way ANOVA with Tukey’s multiple comparisons test (* *p* < 0.05, ** *p* < 0.001, *** *p* < 0.0005, **** *p* < 0.0001). All data shown are representative of three independent experiments.

To characterize the functional role of the circadian clock in CSCs, we used RNA interference (RNAi) to specifically reduce the expression of several circadian clock genes (e.g., *CLOCK, BMAL1, PER1, PER2, CRY1, CRY2, NR1D1, NR1D2*) in 143B CSCs. Significant knockdown of the mRNA expression of each gene was validated by quantitative real-time PCR (qPCR) (Figure 2A).

Once we had confirmed sufficient knockdown, we cultivated the clock gene-depleted cells under 3D culture conditions. Notably, knocking down genes in both the positive (*BMAL1, CLOCK*) and negative (*PER1, PER2, CRY1, CRY2*) limbs of the core clock machinery significantly reduced the number and size of 143B CSC-derived spheroids compared to control siRNA-treated cells (*si-CTL*) (Figure 2B–D, Figure S2A,B). In contrast, individual depletion of *NR1D1* and *NR1D2* (encoding REVERB-α and REVERB-β, respectively) (Figure 2B), which act as BMAL1 repressors in the clock stabilizing loop, had minimal effects on spheroid formation rate and size (Figure 2C,D). To further investigate the mechanism underlying the reduced spheroid formation observed upon clock gene inhibition, immunostaining analysis using a Ki-67 antibody, a marker of cell proliferation, revealed a moderate (e.g., *si-CRY2*) to significant (e.g., *si-BMAL1*, *si-CRY1*) decrease in Ki-67-positive nuclei in clock gene KD cells compared to control (*si-CTL*) cells (Figure S3A,B). Furthermore, live-cell fluorescence imaging with SYTOX necrosis and Annexin V-Cy3 apoptosis staining demonstrated that clock gene inhibition resulted in moderate to significant increases in apoptosis and necrosis in 143B CSC cells (Figure S4A,B). These results align with recent studies showing that *BMAL1* and *CLOCK* downregulation induces cell cycle arrest and apoptosis in glioblastoma stem cells (GSCs) [27] and hepatocellular carcinoma cells [56], while *Cry1*/*Cry2* knockout reduces MYC proto-oncogene expression [57], impairs self-renewal in induced pluripotent stem cells (iPSCs) [58], and promotes apoptosis in cervical cancer cells by suppressing NANOG, inhibiting Signal Transducer and Activator of Transcription 3 (STAT3) signaling, and activating tumor suppressor p53 [59]. These findings suggest the critical role of the core clock machinery in the tumorigenic properties of 143B CSCs, likely through a combination of decreased proliferation and increased cell death.

### 3.3. Impact of Core Clock Gene Knockdown on 143B CSC Migration and Invasion

**Figure 3:**
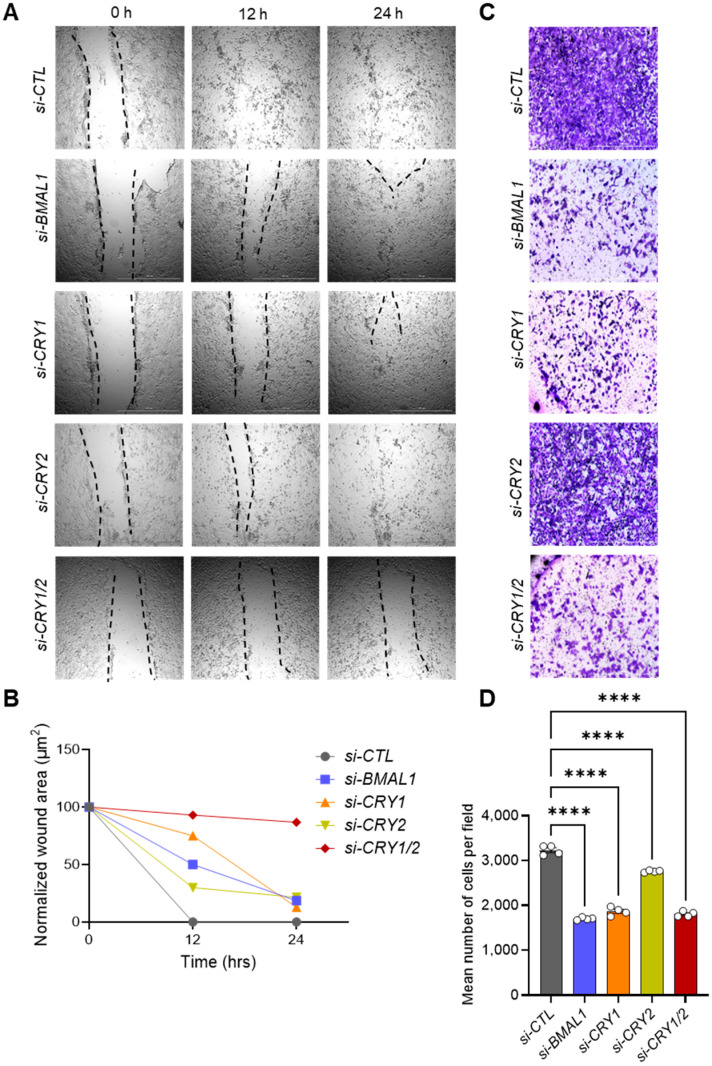
Knockdown of core clock genes reduces the migration and invasion capacities of 143B CSCs. (**A**) Knockdown of core clock genes impairs scratch wound closure. 143B CSCs were transfected with the indicated siRNAs and subjected to scratch wounding assays 48 h post-transfection. Representative images for each condition were captured at 0, 12, and 24 h post-wounding. (**B**) Quantitative analysis of the wound healing migration data shown in (**A**). Wound closure over time was quantified using ImageJ software. (**C**) Knockdown of core clock genes reduces invasive potential. Invasion assays were performed with 143B CSCs 48 h after transfection with the indicated siRNAs. After 24 h, the cells that had invaded to the bottom of the transwell insert were stained with 0.5% crystal violet solution and visualized using brightfield microscopy. Representative images for each condition are shown. (**D**) Quantification of the number of invading cells per field from panel (**C**) was performed using ImageJ and GraphPad Prism software. Statistical significance was determined using one-way ANOVA with Tukey’s multiple comparisons test (**** *p* < 0.0001). All data shown are representative of two independent experiments.

CSCs are frequently associated with the EMT, a process that enhances the motility and invasiveness of cancer cells, traits commonly observed in malignant metastatic progression [13,14,15,16,17]. To further explore the role of core clock genes in CSC behaviors, we performed in vitro scratch wound healing assays to monitor real-time cell migration in live 143B CSCs after siRNA knockdown of *BMAL1* or *CRY1*/*2*, which were selected as representative positive and negative clock regulators, respectively. Interestingly, *si-CTL*-treated cells closed the scratch wound completely within 12 h (Figure 3A,B). However, wound closure was significantly delayed in cells treated with siRNAs targeting *BMAL1* (*si-BMAL1*), *CLOCK* (*si-CLOCK*), *CRY1* (*si-CRY1*), *CRY2* (*si-CRY2*), or both *CRY1* and *CRY2* together (*si-CRY1/2*), with the delay being less pronounced for *CRY2* knockdown alone and most pronounced for *CRY1/2* double knockdown (Figure 3A,B, Figure S5).

To further assess the migration defects associated with core clock gene knockdown, we conducted transwell invasion assays with 143B CSCs transfected with clock gene-targeting siRNAs (Figure 3C,D). Similar to the results of the wound healing assays, knockdown of *BMAL1* and both *CRY1* and *CRY2* together significantly reduced the number of cells that traversed the transwell membrane. Notably, the inhibitory effect of *CRY2* knockdown alone was less pronounced than what was observed with *BMAL1* or *CRY1* knockdown (Figure 3C,D). These findings indicate that BMAL1 and CRY1 play more critical roles than CRY2 in regulating the migratory and invasive properties of 143B CSCs.

### 3.4. Core Clock Genes Differentially Regulate the Expressions of CSC and EMT Markers

**Figure 4:**
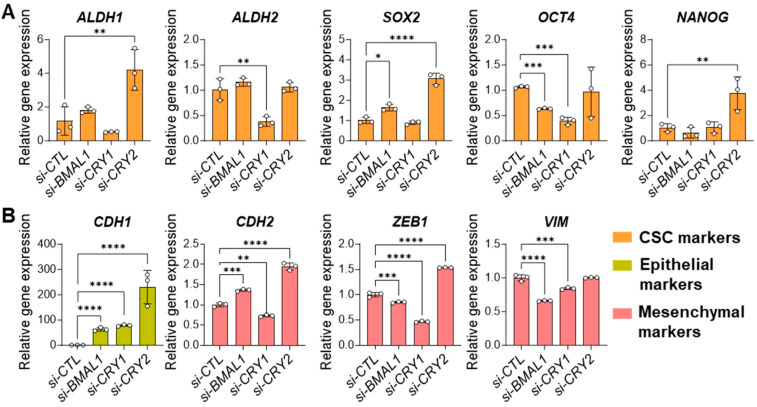
Knockdown of *BMAL1* or *CRY1/2* affects CSC/EMT gene expression in 143B CSCs. (**A**,**B**) qPCR analysis showing the effects of siRNA-mediated knockdown of *BMAL1*, *CRY1*, or *CRY2* (*si-BMAL1*, *si-CRY1*, *si-CRY2*) on the expressions of CSC (**A**) and EMT (**B**) markers compared to control (*si-CTL*-treated) 143B CSCs. Statistical significance was determined by one-way ANOVA with Tukey’s multiple comparisons test (* *p* < 0.05, ** *p* < 0.001, *** *p* < 0.0005, **** *p* < 0.0001). *ALDH1*: Aldehyde Dehydrogenase 1; *ALDH2*: Aldehyde Dehydrogenase 2; *SOX2*: SRY-Box Transcription Factor 2; *OCT4*: Octamer-Binding Transcription Factor 4 (also known as *POU5F1*); *NANOG*: Nanog Homeobox; *CDH1*: Cadherin 1 (commonly referred to as E-Cadherin); *CDH2*: Cadherin 2 (commonly referred to as N-Cadherin); *ZEB1*: Zinc Finger E-Box Binding Homeobox 1; *VIM*: Vimentin. Data shown are representative of three independent experiments.

To determine the molecular mechanisms underlying the effects of core clock gene perturbations on CSC phenotype and behavior, we examined the expressions of well-characterized stem cell (*ALDH1, ALDH2, SOX2, OCT4, NANOG*) and EMT markers (*CDH1, CDH2, ZEB1, VIM*) in core clock gene-depleted 143B CSCs using qPCR analysis. The results showed that among the CSC markers, *ALDH2* and *OCT4* were significantly reduced in *BMAL1*- or *CRY1*-depleted cells compared to controls (Figure 4A), while only *OCT4* was significantly reduced in CLOCK-depleted cells (Figure S6). In contrast, *CRY2* knockdown had little effect on *ALDH2* or *OCT4* expression but significantly upregulated the expression of the other CSC markers (*ALDH1, SOX2, NANOG*) (Figure 4A). We also found that knockdown of *BMAL1, CLOCK,* or *CRY1* upregulated the expression of the epithelial marker (*CDH1*) while downregulating the expression of the mesenchymal markers *CDH2*, *ZEB1,* and *VIM* (Figure 4B, Figure S6). However, *CRY2* knockdown upregulated the expression of most EMT marker genes (*CDH1*, *CDH2*, *ZEB1*) (Figure 4B). This explains, in part, our data showing the weaker inhibitory effect of *CRY2* knockdown, compared to *BMAL1, CLOCK,* or *CRY1* knockdown, on the migratory and invasive capacities of 143B CSCs (Figure 3, Figure S5). Taken together, these findings suggest that components of the core clock machinery have distinct regulatory roles in modulating CSC and EMT marker gene expression that partially contribute to the regulation of CSC properties and behaviors.

### 3.5. Core Clock Genes Regulate Lipid Droplet Biogenesis and Lipid Metabolism Gene Expression in 143B CSCs

**Figure 5:**
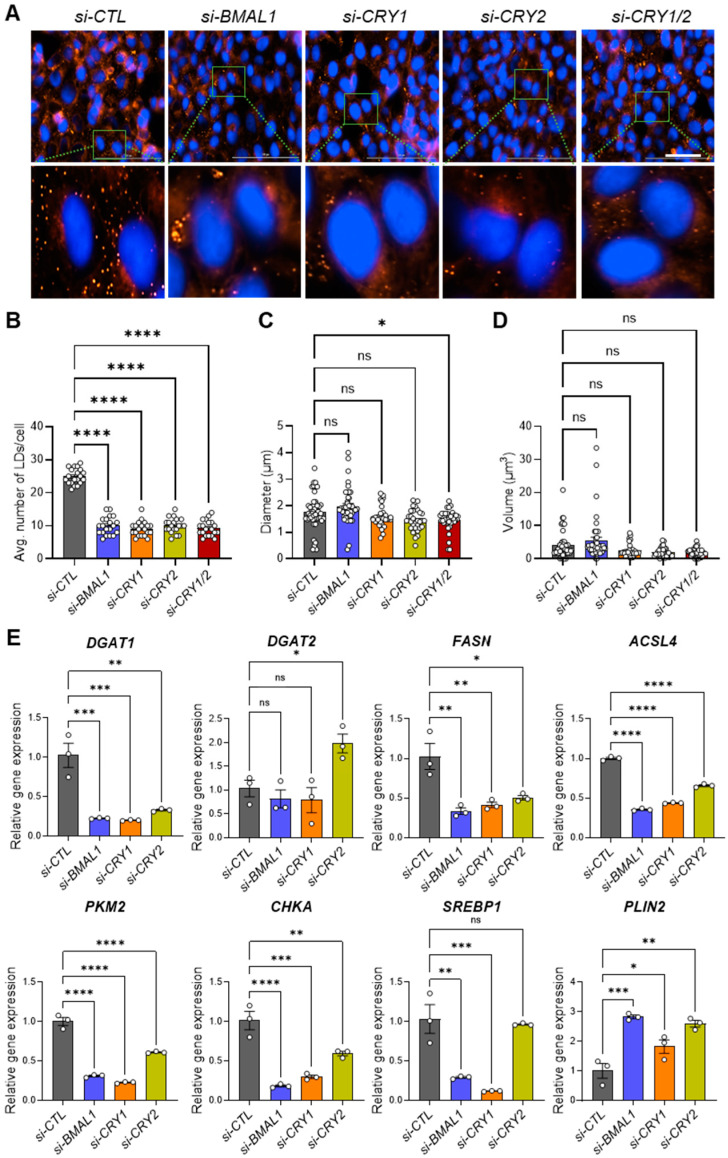
Knockdown of core clock genes disrupts lipid droplet formation by decreasing the expression of genes involved in lipogenesis. (**A**) A total of 15,000 143B CSCs were seeded in each well of a high-content imaging 96-well plate and transfected with control siRNA (*si-CTL*) or siRNAs targeting core clock genes (*si-BMAL1, si-CRY1, si-CRY2, si-CRY1/2*), as indicated. After 48 h, cells were fixed with 4% PFA and stained with Nile Red to visualize lipid droplets (LDs). Nuclei were counterstained with Hoechst. Images were captured using the red fluorescence filter cube (Ex. 531 nm/Em 593 nm) and the DAPI filter cube (Ex. 377 nm/Em 447 nm) on a Cytation 5 multi-mode reader. Representative images are shown. (**B**) LD numbers per cell (n = 20 cells per condition) were quantified from the images in (**A**) using ImageJ software. Each dot represents the LD number in each cell. (**C**,**D**) The diameter (**C**) and volume (**D**) of LDs (n > 40 per condition) were quantified from the images in (**A**) using ImageJ software. (**E**) qPCR analysis was performed to assess the effects of clock gene knockdown on the expression of genes associated with LD biogenesis in 143B CSCs. * *p* < 0.05, ** *p* < 0.001, *** *p* < 0.0005, and **** *p* < 0.0001 by one-way ANOVA with Tukey’s multiple comparisons test; ns, not significant. All of the data shown are representative of three independent experiments. *DGAT1*: Diacylglycerol O-Acyltransferase 1; *DGAT2*: Diacylglycerol O-Acyltransferase 2; *FASN*: Fatty Acid Synthase; *ACSL4*: Acyl-CoA Synthetase Long Chain Family Member 4; *PKM2*: Pyruvate Kinase M2; *CHKA*: Choline Kinase Alpha; *SREBP1*: Sterol Regulatory Element-Binding Protein 1; *PLIN2*: Perilipin 2.

A growing body of evidence suggests that lipid metabolism is intricately linked to cancer progression, with LD accumulation contributing to enhanced CSC tumorigenicity and invasiveness [35,36,37,38,39,40]. To investigate how core clock genes influence LD formation in 143B CSCs, we performed LD staining assays using Nile Red, a lipophilic fluorescent dye commonly used to visualize intracellular LDs [60,61]. Remarkably, knockdown of *BMAL1*, *CLOCK*, *CRY1*, and/or *CRY2* significantly reduced LD numbers in the cells in similar patterns (Figure 5A,B, Figure S7A,B). Further quantification analysis of LD diameters and volumes revealed no substantial differences, except for a moderate but significant reduction in the *CRY1/2* knockdown condition (*si-CRY1/2*) compared to the control (*si-CTL*) (Figure 5C,D, Figure S7C,D).

To uncover the molecular mechanisms behind the suppression of LD formation in 143B CSCs, we conducted qPCR analysis to identify any changes in the expressions of lipid metabolism and LD biogenesis genes (Table 1) [35].

Our results revealed that the expressions of most of the LD-associated genes tested (*DGAT1*, *FASN*, *ACSL4*, *CHKA*, *SREBP1*), with the exception of *DGAT2 and PLIN2*, were significantly reduced in *BMAL1*-, *CLOCK-,* or *CRY1*/*CRY2*-depleted cells compared to cells treated with *si-CTL* (Figure 5E, Figure S7E). Interestingly, *PLIN2*, a key structural component essential for LD integrity, was significantly upregulated in most clock gene knockdown conditions. This suggests that core clock genes predominantly influence lipid synthesis and metabolism without compromising LD structural integrity. Notably, the effects of *CRY2* knockdown were relatively less, as it did not significantly impact *SREBP1* expression or upregulate *DGAT2* (Figure 5E). Importantly, treatment with oleic acid (OA), an inducer of LD formation [74], failed to rescue the reduced invasion capacity of 143B CSCs following clock gene knockdown (*si-CLOCK*, *si-CRY1*), despite the marked increase in invasion under control siRNA conditions (*si-CTL*) (Figure S8). This indicates that core clock genes regulate cell invasion through mechanisms that extend beyond or are independent of LD formation which OA alone cannot restore. Collectively, these findings suggest that components of the core clock machinery are critical for regulating lipid metabolism and LD biogenesis in 143B CSCs, which may be directly linked to clock-regulated CSC/EMT phenotypes and functions.

### 3.6. Core Clock Gene Expression and Prognostic Implications in Human OS Progression

**Figure 6:**
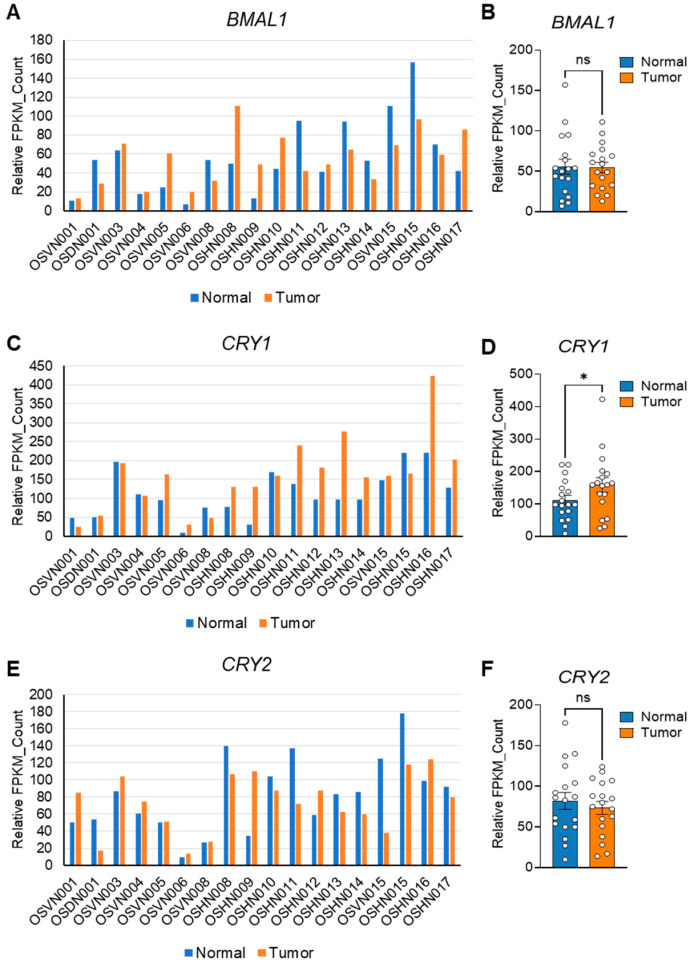
Differential expressions of core clock components in normal and tumor tissues from human OS patients. (**A**,**C**,**E**) Publicly available RNA sequencing data (GSE99671) [48] were used to generate expression profiles for *BMAL1* (**A**), *CRY1* (**C**), and *CRY2* (**E**) in normal (blue) and tumor (orange) tissues harvested from bone samples of 18 individual osteosarcoma (OS) patients. FPKM: Fragments Per Kilobase of Transcript per Million mapped reads. (**B**,**D**,**F**) Bar graphs quantifying the expressions of *BMAL1* (**B**), *CRY1* (**D**), and *CRY2* (**F**) in the normal and tumor tissues shown in (**A**), (**C**), and (**E**), respectively. Statistical significance was determined using one-way ANOVA with Tukey’s multiple comparisons test. * *p* < 0.05. ns: not significant.

The presence of CSCs and EMT phenotypes is closely associated with malignant tumor progression and poor prognosis in clinical patients [75,76,77]. Based on our in vitro data showing the critical role of core clock components in CSC/EMT signatures, we performed integrated transcriptomic analyses on publicly available datasets to investigate the impacts of these factors on human OS progression and prognosis. To examine the differential expressions of *BMAL1*, *CLOCK*, *CRY1*, and *CRY2* in normal versus tumor OS tissues, we analyzed previously published RNA sequencing data (GSE99671) from 18 OS patients [48]. Interestingly, despite individual variability (Figure 6A,C,E, Figure S9A), *BMAL1* expression remained unchanged, while CRY1 was significantly upregulated, *CRY2* showed a downregulation trend (Figure 6B,D,F), and *CLOCK* exhibited an upregulation trend in tumors compared to normal tissues, though not statistically significant (Figure S9B).

**Figure 7:**
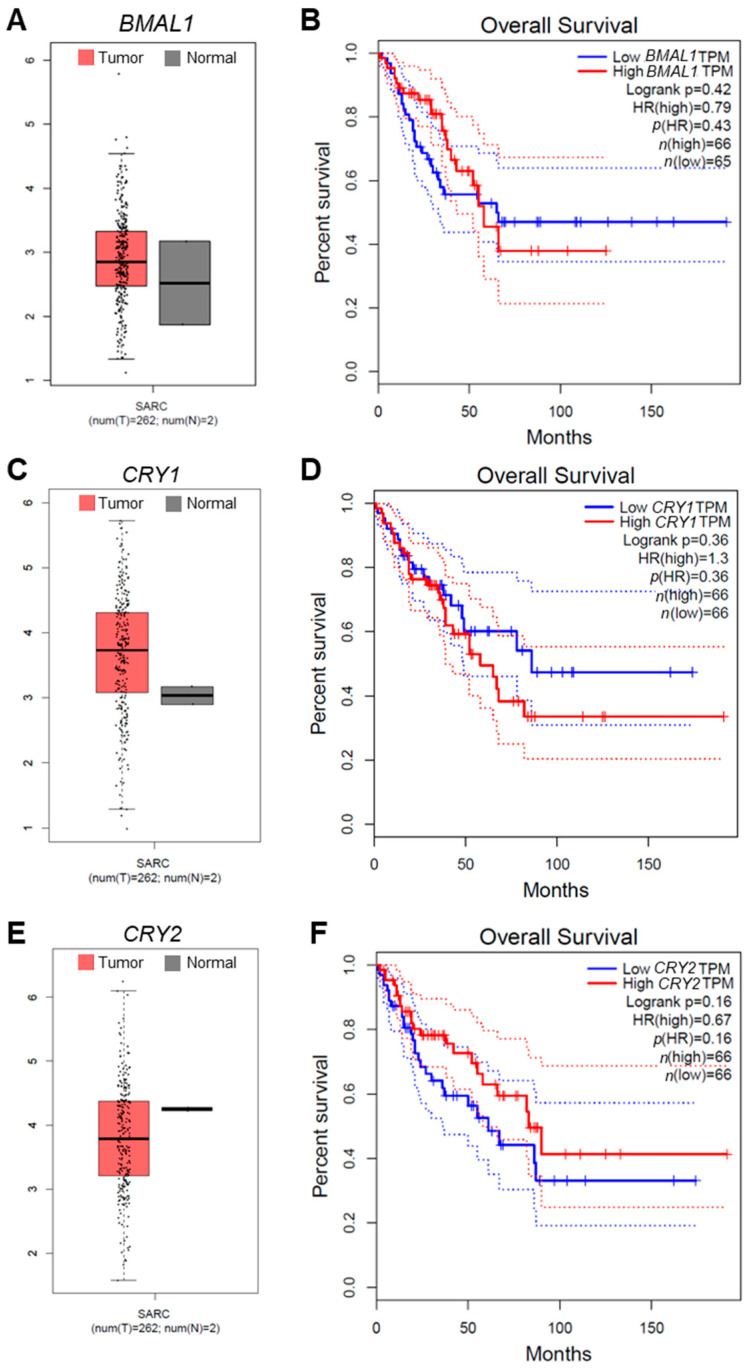
The correlation of core clock gene expression with sarcoma patient prognosis and outcomes. (**A**,**C**,**E**) Box plot analysis of the expressions of *BMAL1* (**A**), *CRY1* (**C**), and *CRY2* (**E**) in tumor (T, crimson) and normal (N, dark gray) tissues from human sarcoma (SARC) patients. Plots were generated with data from Gene Expression Profiling Interactive Analysis (GEPIA; http://gepia.cancer-pku.cn/; accessed on 1 June 2024). (**B**,**D**,**F**) Survival curves for SARC patients based on the expression levels of *BMAL1* (**B**), *CRY1* (**D**), and *CRY2* (**F**) were generated using GEPIA. Analysis of the survival data was performed with the Cox proportional hazard model, and Kaplan–Meier (KM) curve parameters were applied to evaluate the significance of gene expression on patient outcomes. Each KM plot includes the hazard ratio (HR), Cox model *p*-value, and logrank *p*-value. TPM, Transcripts Per Million.

Similar to our OS gene expression analysis, complementary analysis of sarcoma (SARC) patient data from Gene Expression Profiling Interactive Analysis (GEPIA) revealed that *CRY1* and *CLOCK* exhibited higher expression in tumors compared to *BMAL1* or *CRY2* (Figure 7A,C,E, Figure S9C). Furthermore, corresponding survival analysis showed that elevated *CRY1* and *CLOCK* expression in tumor tissues was strongly correlated with poorer patient prognosis, compared to what was observed for *BMAL1* or *CRY2* (Figure 7B,D,F, Figure S9D).

**Figure 8:**
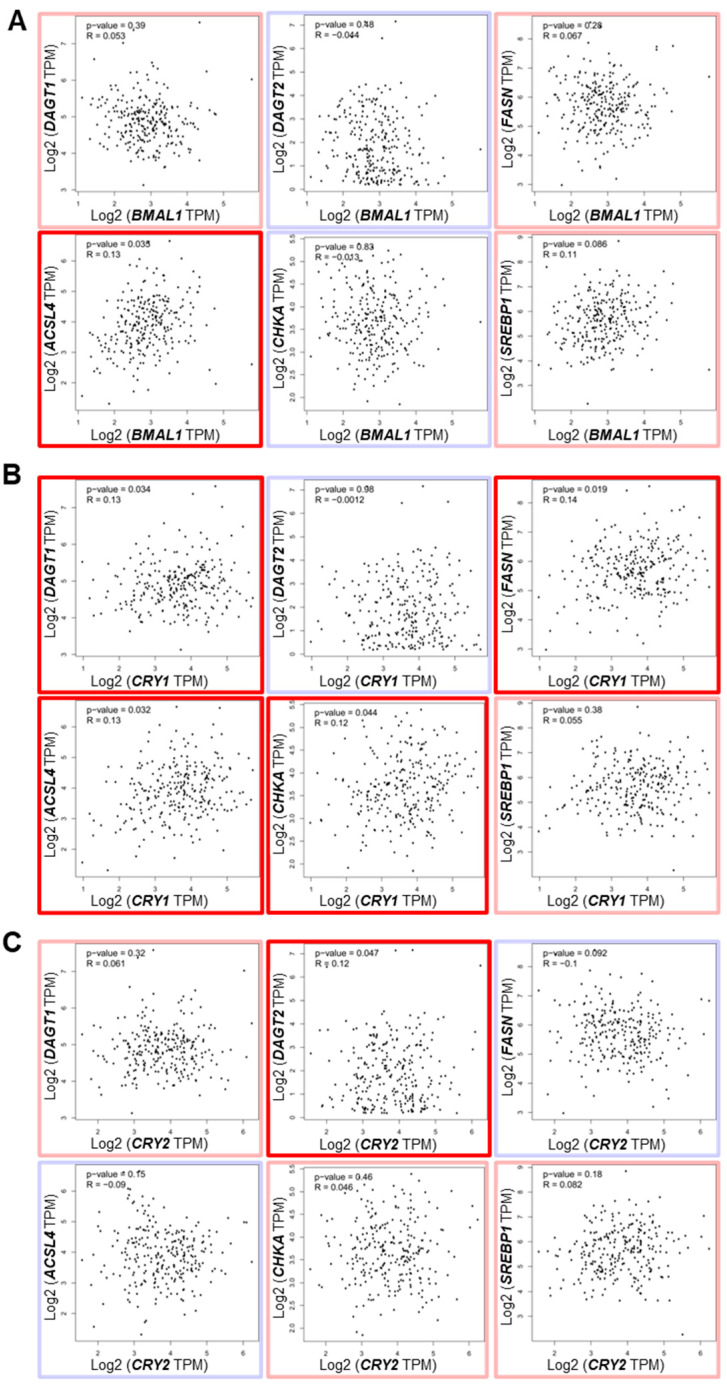
The correlation of core clock component expression with lipid droplet biogenesis-associated gene expression in human OS patients. (**A–C**) Correlations between the expressions of *BMAL1* (**A**), *CRY1* (**B**), and *CRY2* (**C**) and genes associated with lipid droplet (LD) biogenesis (*DGAT1*, *DGAT2*, *FASN*, *ACSL4*, *CHKA*, *SREBP1*) in human osteosarcoma patient samples were determined. Data were sourced from the Gene Expression Profiling Interactive Analysis (GEPIA; http://gepia.cancer-pku.cn/; accessed on 1 June 2024). Genes with positive and negative correlations, based on purity-adjusted partial Spearman’s rho values, are highlighted with light red and light blue boxes, respectively. Genes with significantly positive correlations (*p* < 0.05) are marked with bold red boxes.

In additional gene correlation analysis, *CRY1* was significantly positively associated with several lipid metabolism/LD-associated genes, including *DGAT1*, *FASN*, *ACSL4*, and *CHKA*, which was more than what was observed for *BMAL1*, *CLOCK*, and *CRY2* (Figure 8A–C, Figure S10, bold red boxes).

While these correlations were statistically significant, the correlation coefficients were relatively low (r = 0.12–0.14) with modest *p*-values (*p* = 0.02–0.04), suggesting that these associations, though present, may be influenced by the large sample size. Notably, *CRY2* exhibited a unique positive correlation with *DGAT2*, contrasting with the negative correlation of *DGAT2* with *BMAL1* and *CRY1* (Figure 8C). This observation is reminiscent of our in vitro data showing the opposing effects of knockdown of *CRY2* and *BMAL1*/*CRY1* on *DGAT2* expression (Figure 5C). Collectively, these findings indicate that CRY1 may serve as a prominent pro-tumorigenic factor among the core clock components, potentially contributing to OS progression and prognosis by modulating lipid metabolism and LD biogenesis pathways.

## 4. Discussion

It has been suggested that circadian functions are often impaired or lost in cancer cells, facilitating uncontrolled tumor proliferation and growth [78,79]. Previous bulk analyses of tumor cells in monolayer cultures or tumor tissues grafted in mice have underscored the tumor-suppressive roles of clock genes [78,79]. However, emerging studies have shown that tumorigenic CSC populations, particularly glioblastoma stem cells (GSCs) and leukemia stem cells (LSCs), exhibit robust circadian rhythms under cultured conditions [26,27,28]. Furthermore, circadian molecular functions involving the core clock transcription factors BMAL1 and CLOCK have been proven to be required for the survival of CSC populations in brain and blood cancers [26,27,28]. Aligning with these findings, our study shows that human 143B OS CSCs retain notable circadian rhythms (Figure 1), with both positive (i.e., *BMAL1, CLOCK*) and negative (i.e., *CRY1/2, PER1/2*) primary loop components of the core clock machinery playing crucial roles in regulating the tumorigenic and/or invasive properties of these cells by modulating multiple genes related to CSC/EMT processes and lipid metabolism/LD biogenesis (Figure 2, Figure 3, Figure 4 and Figure 5, Figures S2–S8). In addition, gene correlation and outcome analyses of human OS patient data identified CRY1 as a significant tumorigenic factor driving malignant OS progression and poor survival, likely through its regulation of lipid metabolism genes (Figure 6, Figure 7 and Figure 8).

Emerging studies have suggested the circadian regulation of lipid metabolism and LD formation, linking these processes to metabolic disorders, including cancer. [41,42,80]. In line with this, our study highlights a close interplay between the circadian molecular clock, CSC/EMT, and lipid metabolism. Supporting the critical role of core clock components in LD biogenesis that we observed (Figure 5, Figure S7), a recent study using the HepG2 hepatocellular carcinoma model demonstrated that disrupting the molecular clock by knocking out *BMAL1* via CRISPR/Cas9 significantly reduced lipid metabolism and LD formation by impairing the expression of lipid-synthesizing enzymes, such as *CHKA*, *PCYT2*, and *LPIN1* [44]. Another study reported a positive feedback loop between the EMT transcription factor ZEB2 and the lipid metabolic enzyme ACSL4 that enhances LD accumulation and fatty acid oxidation to drive breast cancer metastasis [81]. Given the observed positive interactions between core clock components (e.g., *BMAL1*, *CLOCK*, *CRY1*) and EMT markers (e.g., *ZEB1*, *VIM*), as well as LD factors (e.g., *DGAT1*, *ACSL4*) (Figure 4, Figure 5, Figure 8, Figures S6 and S7), we speculate that the circadian clock orchestrates a positive feedback loop between CSC/EMT pathways and lipid metabolism. Further studies are warranted to elucidate the detailed mechanisms underlying these interactions. Additionally, while our analysis of a human patient dataset revealed statistically significant positive correlations between CRY1 and several lipid metabolism genes, the low r values (0.12–0.14) and modest p-values (0.02–0.04) indicate that these associations are weak (Figure 8). This finding, likely influenced by the large dataset, warrants cautious interpretation. Further studies with larger cohorts and complementary functional assays are needed to confirm the biological relevance of these correlations.

Notably, previous studies on the functional roles of CRY1 and CRY2 in human OS cells (i.e., U-2OS, HOS) showed that these factors have tumor-suppressive functions, with *CRY1* or *CRY2* knockdown enhancing cell proliferation and migration [82,83]. In contrast, our study with 143B OS CSCs revealed that both CRY1 and CRY2 exert pro-tumorigenic and pro-invasive functions in CSC populations, with CRY1 playing a more prominent role, as evidenced by the greater impact of *CRY1* knockdown on CSC/EMT-associated phenotype and gene expression (Figure 2, Figure 3 and Figure 4). The observed discrepancy between our findings and those of previous studies may be due to differences in cell type (i.e., U-2OS/HOS vs. 143B) or cellular context (CSCs vs. non-CSCs). Supporting our findings, several cancer model studies have highlighted CRY1′s tumor-promoting role and its potential as a prognostic marker. For instance, previous studies have shown that CRY1 overexpression correlates with tumor progression and poor prognosis in patients with colorectal cancer [84], gastric cancer [85], and chronic lymphocytic leukemia [86]. Additionally, mirroring our findings regarding CRY1′s regulation of CSC phenotype and marker expression, the combined overexpression of CRY1 and NANOG in cervical cancer has been associated with poor survival outcomes and enhanced prediction of chemoradiation responses, with *CRY1* knockdown inducing apoptosis and reducing NANOG expression [59]. A more recent study also reported a pro-tumorigenic role for CRY1 in prostate cancer development and progression [87]. Along with our results, these findings suggest that targeting CRY1 may have therapeutic potential in preventing CSC/EMT processes, the malignant progression associated with these processes, and poor prognosis in multiple cancer types, including OS.

Importantly, increasing evidence suggests that there is dynamic crosstalk between CSCs and immune cells within the tumor microenvironment that plays crucial roles in tumor progression, recurrence, and therapy responses [88,89,90]. Based on our human OS patient sample analysis showing the pre-eminence of CRY1 over other core clock genes (i.e., BMAL1, CRY2) as a potential prognostic marker (Figure 6, Figure 7 and Figure 8), we cannot rule out the possibility that, in addition to the differential intrinsic metabolic functions of core clock components in CSCs, their interactions with tumor–immune microenvironmental factors such as pro-tumoral or anti-tumoral immune and stromal cells may also contribute to the differential roles of these core clock components in OS progression and prognosis in human patients. Supporting this notion, a recent preprint [91] has reported that CRY1 is a key NANOG target in high-NANOG melanoma and cervical cancer cells, promoting CSC-like properties and resistance to T-cell killing by stabilizing Cyclin A and myeloid cell leu-kemia sequence 1 (MCL1) while suppressing C-X-C motif chemokine ligand 10 (CXCL10) via HDAC1-mediated repression [91]. Correlation analysis in TCGA datasets, including sarcoma, linked CRY1 activity to stemness, immune evasion, and poor T-cell infiltration [91]. Moreover, CRY1 inhibition was shown to synergize with programmed death-1 (PD-1) blockade and adoptive T-cell transfer, converting immune-resistant tumors into immune-sensitive ones [91]. These findings suggest that CRY1 may serve as a critical nexus linking tumor-intrinsic pathways with immune evasion, warranting further investigation into whether similar mechanisms operate in osteosarcoma. This underscores the need for additional studies using in vivo animal and clinical model systems to better understand these interactions.

## Figures and Tables

**Table 1:** Roles of lipid droplet biogenesis and lipid metabolism-associated genes.

Genes	Functions	References
*DGAT1*	Catalyzes the final step of triacylglycerol (TAG) synthesis by esterifying diacylglycerol (DAG) with fatty acids, a crucial process for lipid droplet (LD) assembly.	[62]
*DGAT2*	Works alongside DGAT1 to synthesize triglycerides and facilitate lipid droplet accumulation, influencing tumor cell growth and migration.	[62]
*FASN*	Drives de novo fatty acid synthesis, providing essential substrates for TAG production and LD formation, and contributes to cancer cell lipid metabolism.	[63]
*ACSL4*	Activates polyunsaturated fatty acids (PUFAs), supports lipid droplet biogenesis, and is often overexpressed in tumor cells to enhance lipid storage related to cancer progression.	[64,65]
*PKM2*	Regulates anabolic metabolism by promoting lipid synthesis, links glycolysis with fatty acid production, and influences lipid droplet dynamics in proliferating cancer cells.	[66]
*CHKA*	Involved in phosphatidylcholine synthesis, contributing to membrane biogenesis and the stabilization of lipid droplets, which supports cancer cell viability.	[37,67,68]
*SREBP1*	A master regulator of lipid metabolism that activates genes involved in fatty acid and cholesterol synthesis, facilitating LD formation and promoting cancer cell proliferation.	[35,69]
*PLIN2*	PLIN2 is a ubiquitously expressed lipid droplet protein essential for lipid sequestration and TG homeostasis. It stabilizes lipid droplets, potentially limiting their accessibility for autophagosome biogenesis and influencing key enzymes involved in lipid metabolism.	[70,71,72,73]

## Data Availability

The original contributions presented in this study are included in the article/Supplementary Materials. Further inquiries can be directed to the corresponding author.

## Supplementary Materials

The following supporting information can be downloaded at https://www.mdpi.com/article/10.3390/cells14070517/s1: Figure S1. A comparison of the spheroid formation capacities of different human OS cell lines under 3D culture conditions. Figure S2. Knockdown of core clock genes reduces spheroid formation in 143B CSCs. Figure S3. Knockdown of core clock genes affects cell proliferation in 143B CSCs. Figure S4. Knockdown of core clock genes impacts cell viability in 143B CSCs. Figure S5. Knockdown of *CLOCK* reduces the migration capacities of 143B CSCs. Figure S6. Knockdown of *CLOCK* affects CSC/EMT gene expression in 143B CSCs. Figure S7. Knockdown of *CLOCK* disrupts lipid droplet formation by influencing the expression of genes involved in lipogenesis. Figure S8. The effect of oleic acid treatment on the invasion capacity of 143B CSCs following core clock gene knockdown. Figure S9. Differential expressions of *CLOCK* in normal and tumor tissues from human OS patients. Figure S10. The correlation of *CLOCK* with lipid droplet biogenesis-associated gene expression in human OS patients.

## Appendix A

**Table A1:** qPCR primers for circadian clock genes.

Primers	Forward Sequence	Reverse Sequence
*CLOCK*	ATGGGCCAGGTGGTGACTGCAT	TGACCCAGCCACCGCAACAAT
*BMAL1*	CCAGAGGCCCCTAACTCCTC	TGGTCTGCCATTGGATGATCT
*PER1*	CAGTGCTCCTGTTCCTGCATC	CCCGCCAACTGCAGAATCT
*PER2*	AATGCCGATATGTTTGCGGT	GCATCGCTGAAGGCATCTCT
*CRY1*	CAACCTCCATTCATCTTTCC	CTCATAGCCGACACCTTC
*CRY2*	TCCCAAGGCTGTTCAAGGAAT	TGCATCCCGTTCTTTCCCAAA
*NR1D1*	CCGTGACCTTTCTCAGCATGA	GACTGTCTGGTCCTTCACGTTG
*NR1D2*	ACTCCAGCAGTGAAAGAAGTGGTGG	GACTGTCTGGTCCTTCACGTTG

**Table A2:** qPCR primers for CSC/EMT marker genes.

Primers	Forward Sequence	Reverse Sequence
*ALDH1*	TGTTAGCTGATGCCGACTTG	TTCTTAGCCCGCTCAACACT
*ALDH2*	CCATTGGAGTGTGTGGACAG	GATGAGGGCTCCCATGTAGA
*SOX2*	CACATGAACGGCTGGAG	CTGGTCATGGAGTTGTACTG
*OCT4*	GGGAAGGTATTCAGCCAAAC	AGAACCACACTCGGACC
*NANOG*	AACTCTCCAACATCCTGAAC	GTAGGAAGAGTAAAGGCTGG
*CDH1*	ATTCTGATTCTGCTGCTCTTG	ATTCTGATTCTGCTGCTCTTG
*CDH2*	GTGCATGAAGGACAGCCTCT	CCACCTTAAAATCTGCAGGC
*ZEB1*	GATGATGAATGCGAGTCAGATGC	CTGGTCCTCTTCAGGTGCC
*VIM*	ACACCCTGCAATCTTTCAGACA	GATTCCACTTTGCGTTCAAGGT

**Table A3:** qPCR primers for LD biogenesis and lipid metabolism-associated genes.

Primers	Forward Sequence	Reverse Sequence
DGAT1	TATTGCGGCCAATGTCTTTGC	CACTGGAGTGATAGACTCAACCA
DGAT2	TCTCACGGAGGACCTGC	CACCAGCCAAGTGAAGTAGAG
FASN	CATCCAGATAGGCCTCATAGA	CTCCATGAAGTAGGAGTGGAA
ACSL4	CATCCCTGGAGCAGATACTCT	TCACTTAGGATTTCCCTGGTCC
PKM2	ATCGTCCTCACCAAGTCTGG	GAAGATGCCACGGTACAGGT
CHKA	GGCCAAGATCTCATCTATTGAA	TGGTGGAAATAGGCATCAAACC
SREBP1	CCATGGATTGCACTTTCGAA	GGCCAGGGAAGTCACTGTCTT
PLIN2	GAGTGGAAAAGGAGCATTGGA	CCTTGGATGTTGGACAGGAG
