# Supplementary material for: Core Molecular Clock Factors Regulate Osteosarcoma Stem Cell Survival and Behavior via CSC/EMT Pathways and Lipid Droplet Biogenesis
Source: Cells. 2025 Mar 31;14(7):517. doi: 10.3390/cells14070517 (PMC11988071; doi:10.3390/cells14070517)
Supplement: Supplementary file 1 [file cells-14-00517-s001.zip › cells-3465657-supplementary.pdf]

Supplementary Figures

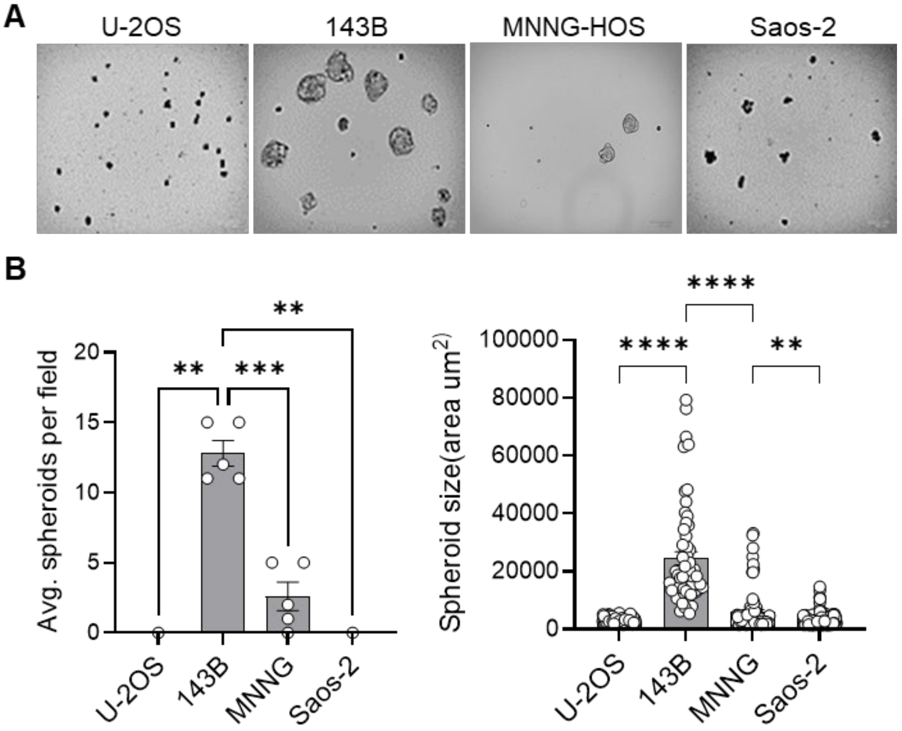

Figure S1. Comparing spheroid forming capacity of different human OS cell lines in 3D culture conditions. (**A**) Representative brightfield images of spheroids were captured after one week of culture, where human osteosarcoma (OS) cell lines (U-2OS, 143B, MNNG, and Saos-2) were seeded at 10,000 cells per well in 0.5% methylcellulose-containing 3D culture media on a 6-well low-attachment plate. (**B**) Quantitative analysis of spheroid numbers and sizes shown in (A) was performed using ImageJ and GraphPad Prism software. Statistical significance was determined by one-way ANOVA with Tukey's multiple comparisons test (*$p < 0.05$, **$p < 0.001$, ****$p < 0.0001$).

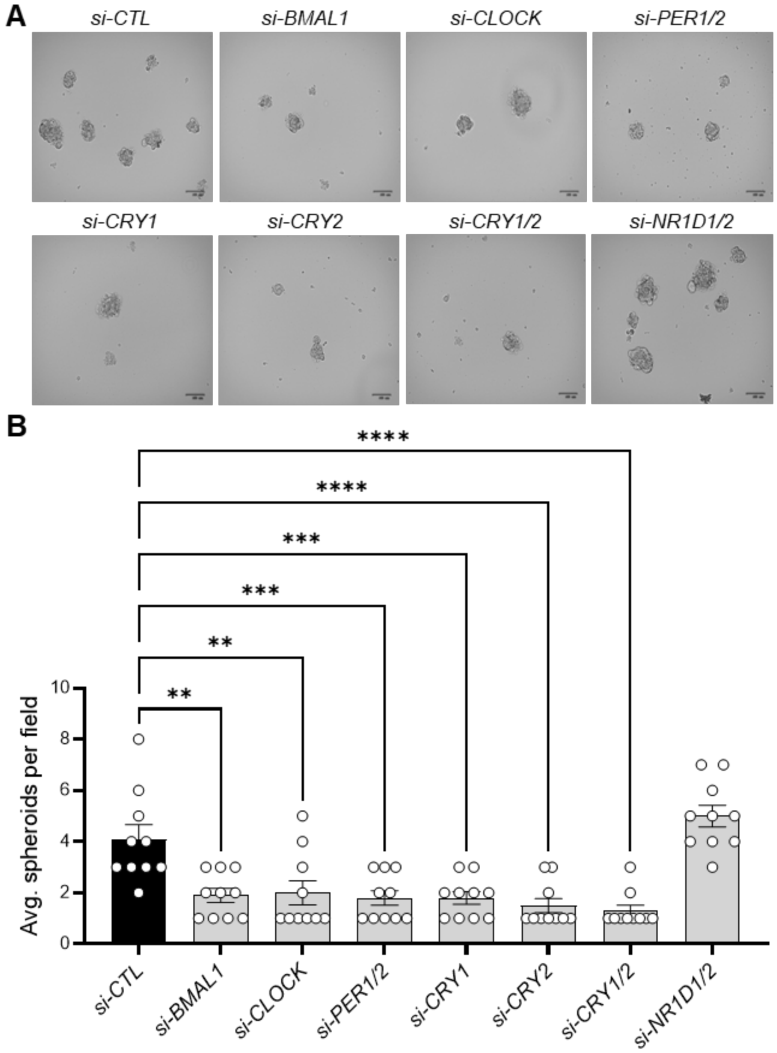

Figure S2. Knockdown of core clock genes reduces spheroid formation in 143B CSCs. (**A**) Knockdown of core clock genes reduces spheroid formation. 143B CSCs were transfected with the indicated siRNAs for 48 hours and then seeded for spheroid formation at a density of 10,000 cells per well in low-attachment 6-well plates. After one week in culture, brightfield images (n > 5) of the siRNA-treated spheroids were captured. Representative images are shown. (**B**) The numbers of the spheroids in (A) were quantified using ImageJ software. Statistical significance was determined by one-way ANOVA with Tukey's multiple comparisons test (**p < 0.001, ***p < 0.0005, ****p < 0.0001). All data shown are representative of three independent experiments.

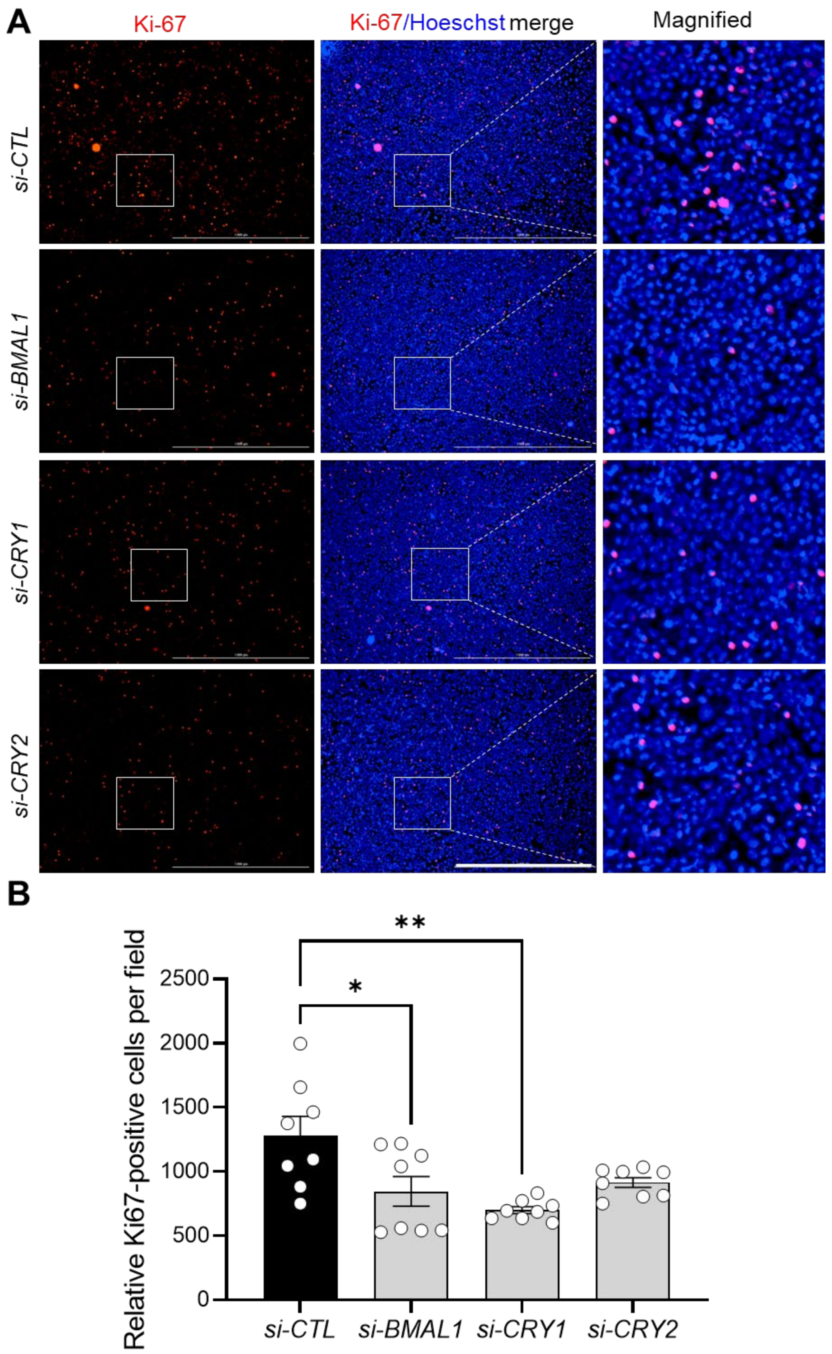

Figure S3. Knockdown of core clock genes affects cell proliferation in 143B CSCs. (**A**) 143B CSCs were seeded in a high-content imaging 96-well plate and transfected with control (*si-CTL*) or the specific clock gene siRNAs (*si-BMAL1*, *si-CRY1*, *si-CRY2*) as indicated. Forty-eight hours post-transfection, cells were fixed with 4% PFA, permeabilized with Triton X-100, and incubated with a Ki-67 antibody (red). After secondary antibody incubation, cells were counterstained with Hoechst nuclear dye (blue). Immunofluorescence images were captured using fluorescence microscopy (Cytation 5) with RFP and DAPI filters. Representative images taken at 4×

magnification are shown, with magnified regions highlighted using rectangular boxes for each condition. (**B**) Quantification of Ki-67-positive nuclei from images (n = 8 per condition) in (A) was performed using Cytation 5 Gen5 software. Statistical significance was determined using two-way ANOVA with Bonferroni's multiple comparisons test (*p < 0.05, *p < 0.01, **p < 0.005).

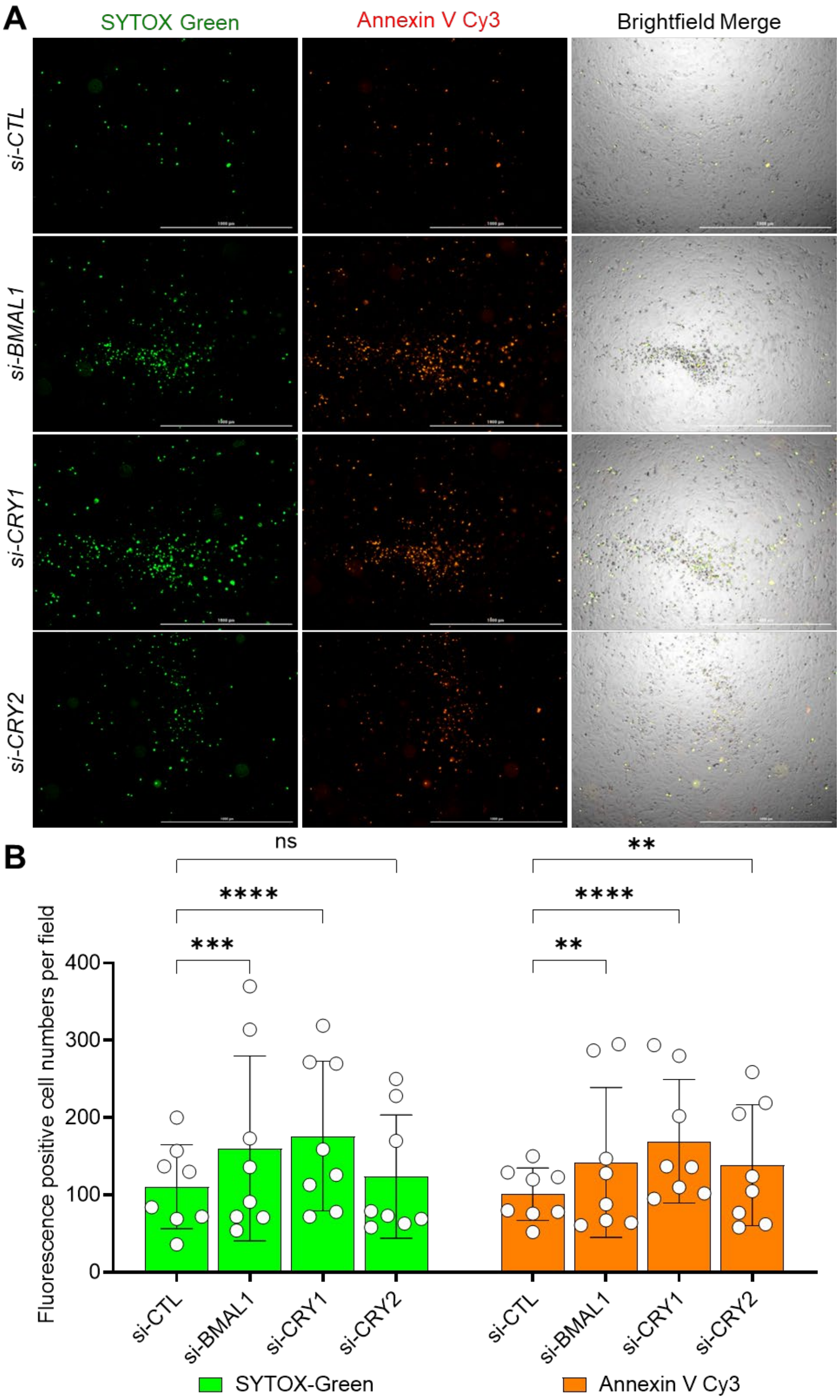
A
SYTOX Green
Annexin V Cy3
Brightfield Merge
si-CTL
si-BMAL1
si-CRY1
si-CRY2
B
ns
**
****
****
***
**
Fluorescence positive cell numbers per field
400
300
200
100
0
si-CTL
si-BMAL1
si-CRY1
si-CRY2
si-CTL
si-BMAL1
si-CRY1
si-CRY2
SYTOX-Green
Annexin V Cy3

Figure S4. Knockdown of core clock genes impacts cell viability in 143B CSCs. (**A**) 143B CSCs were seeded in a high-content imaging 96-well plate and transfected with control (*si-CTL*) or specific clock gene siRNAs (*si-BMAL1*, *si-CRY1*, *si-CRY2*) as indicated. After 48 hours, cells were stained with SYTOX Green (necrosis) and Annexin V-Cy3 (apoptosis) and imaged using Cytation 5 with fluorescence filter sets for SYTOX Green (Ex. 488 nm/Em. 530 nm) and Annexin V-Cy3 (Ex. 543 nm/Em. 570 nm). Representative images are shown. (**B**) Fluorescence-positive cells from (A) were quantified using Cytation 5 Gen5 software (n = 8 per condition). Statistical significance was determined by two-way ANOVA with Tukey's multiple comparisons test (**p < 0.005, ***p < 0.001, ****p < 0.0001).

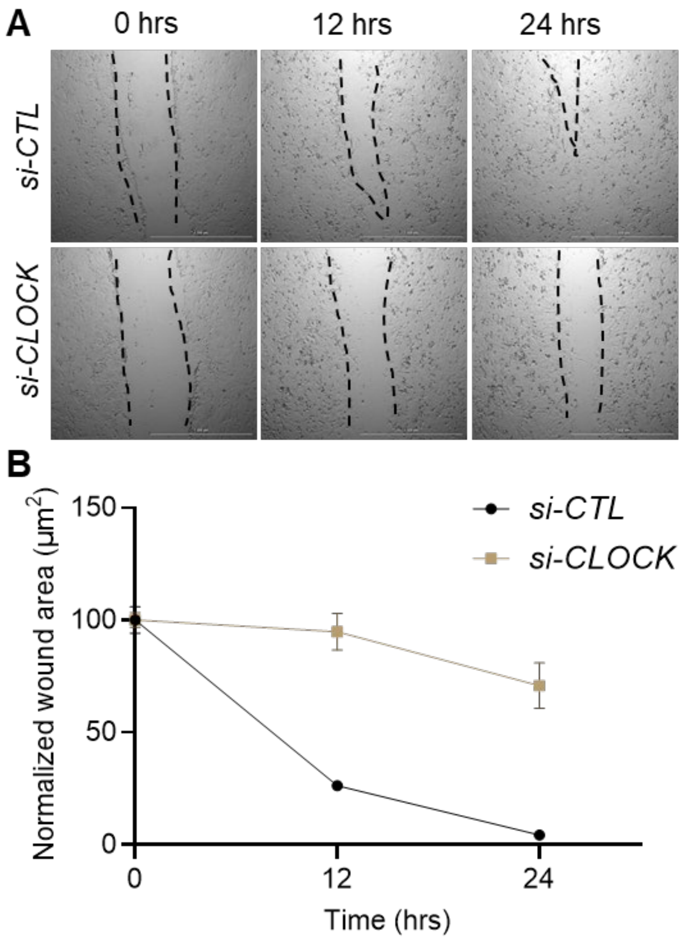

Figure S5. Knockdown of CLOCK reduces the migration capacities of 143B CSCs. (**A**) Knockdown of CLOCK gene impairs scratch wound closure. 143B CSCs were transfected with *CLOCK* targeting siRNA (*si-CLOCK*) and subjected to scratch wounding assays 48 hours post-transfection. Representative images for each condition were captured at 0-, 12-, and 24-hours post-wounding. (**B**) Quantitative analysis of the wound healing migration data shown in (A). Wound closure over time was quantified using ImageJ software. (C) Knockdown of core clock genes reduces invasive potential. The data shown are representative of two independent experiments.

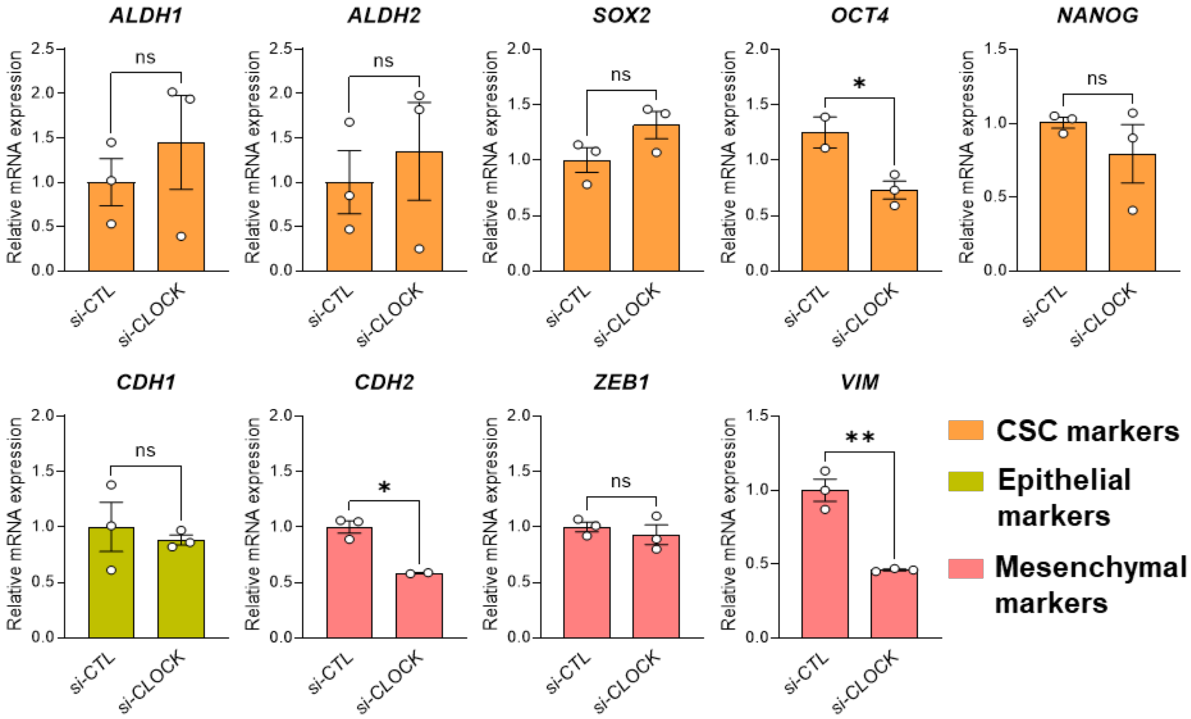

Figure S6. Knockdown of CLOCK affects CSC/EMT gene expression in 143B CSCs. qPCR analysis showing the effects of siRNA-mediated knockdown of *CLOCK* (*si-CLOCK*) on the expressions of CSC and EMT markers compared to control (*si-CTL*-treated) 143B CSCs. Statistical significance was determined using a Student's t-test (*p < 0.05, **p < 0.005). ALDH1: Aldehyde Dehydrogenase 1, ALDH2: Aldehyde Dehydrogenase 2, SOX2: SRY-Box Transcription Factor 2, OCT4: Octamer-Binding Transcription Factor 4 (also known as POU5F1), NANOG: Nanog Homeobox, CDH1: Cadherin 1 (commonly referred to as E-Cadherin), CDH2: Cadherin 2 (commonly referred to as N-Cadherin), ZEB1: Zinc Finger E-Box Binding Homeobox 1, VIM: Vimentin. Data shown are representative of three independent experiments.

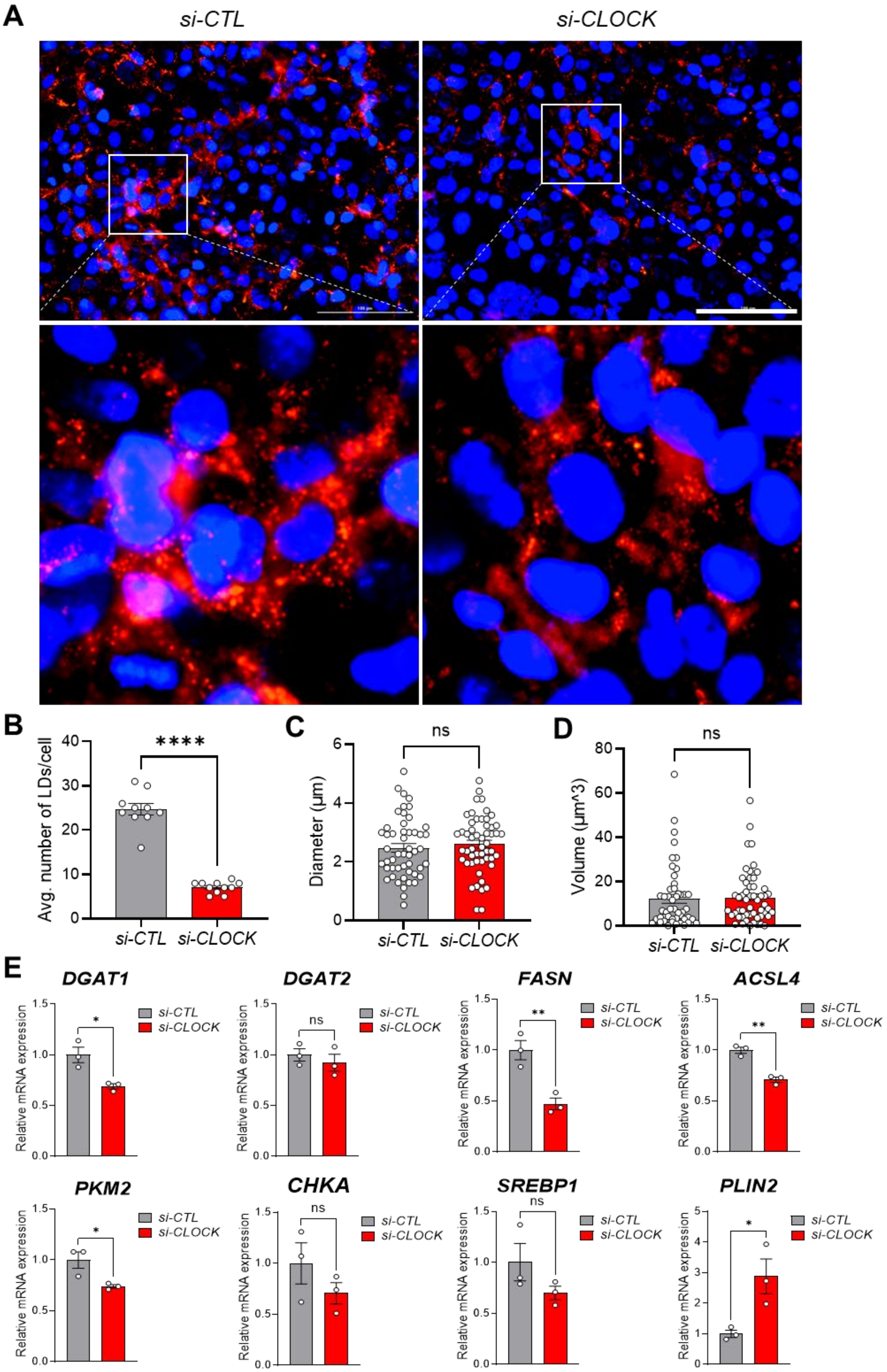
A
si-CTL
si-CLOCK
B
Avg. number of LDs/cell
****
si-CTL
si-CLOCK
C
Diameter (µm)
ns
si-CTL
si-CLOCK
D
Volume (µm^3)
ns
si-CTL
si-CLOCK
E
DGAT1
DGAT2
FASN
ACSL4
PKM2
CHKA
SREBP1
PLIN2
Relative mRNA expression
si-CTL
si-CLOCK
ns
*
**

Figure S7. Knockdown of *CLOCK* disrupts lipid droplet formation by influencing the expression of genes involved in lipogenesis. (**A**) A total of 15,000 143B CSCs were seeded in each well of a high-content imaging 96-well plate and transfected with control siRNA (*si-CTL*) or siRNA targeting *CLOCK* (*si-CLOCK*). After 48 hours, cells were fixed with 4% PFA and stained with Nile Red to visualize lipid droplets (LDs). Nuclei were counterstained with Hoechst. Images were captured using the red fluorescence filter cube (Ex. 531 nm/Em 593 nm) and the DAPI filter cube (Ex. 377 nm/Em 447 nm) on a Cytation 5 multi-mode reader. Representative images are shown. Scale bar: 100 µm. (**B**) LD numbers per cell (n = 10 cells per condition) were quantified from the images in (A) using ImageJ software. Each dot represents the LD number in each cell. (**C, D**) The diameter (C) and volume (D) of LDs (n > 40 per condition) were quantified from the images in (A) using ImageJ software. (**E**) qPCR analysis was performed to assess the effects of *CLOCK* knockdown (*si-CLOCK*) on the expression of genes associated with LD biogenesis in 143B CSCs. Statistical significance was determined using a Student's t-test ($^*p < 0.05$, $^{**}p < 0.005$, $^{****}p < 0.0001$). DGAT1: Diacylglycerol O-Acyltransferase 1, DGAT2: Diacylglycerol O-Acyltransferase 2, FASN: Fatty Acid Synthase, ACSL4: Acyl-CoA Synthetase Long Chain Family Member 4, PKM2: Py-ruvate Kinase M2, CHKA: Choline Kinase Alpha, SREBP1: Sterol Regulatory Element-Binding Protein 1. PLIN2: Perilipin 2.

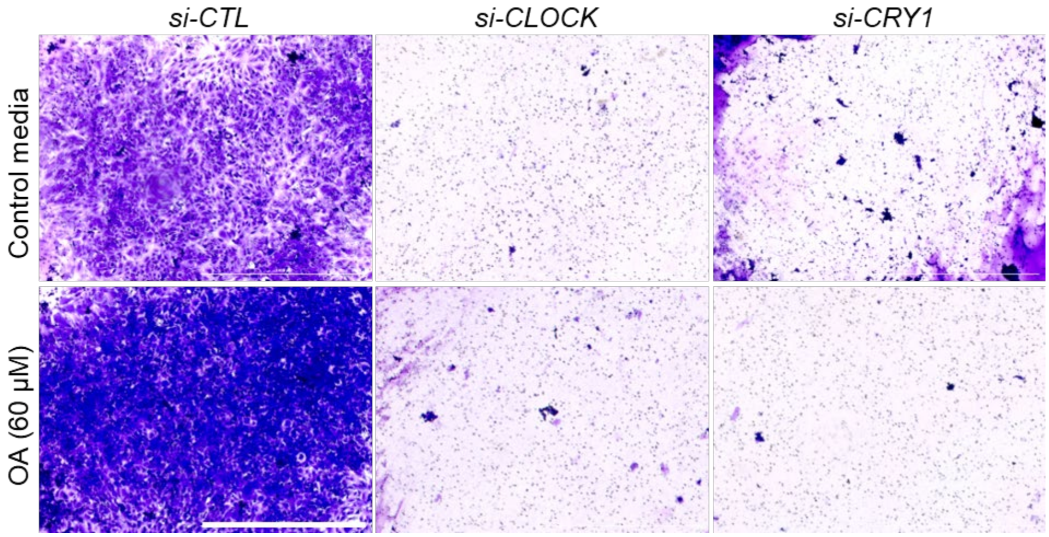

Figure S8. Effect of oleic acid treatment on the invasion capacity of 143B CSCs following core clock gene knockdown. 143B CSC cells were transfected with control (*si-CTL*), CLOCK (*si-CLOCK*), or CRY1 (*si-CRY1*) siRNA and treated with either oleic acid (OA, 60 μM) or control media for 24 hours. After incubation, Matrigel-coated transwell inserts were prepared, and cells were seeded onto the inserts with 10% FBS as a chemoattractant, followed by a 24-hour incubation. The inserts were then fixed with 4% PFA, stained with 0.5% crystal violet, and visualized using a Cytation Imaging Reader. Representative images are shown. Scale bar: 1000 μm.

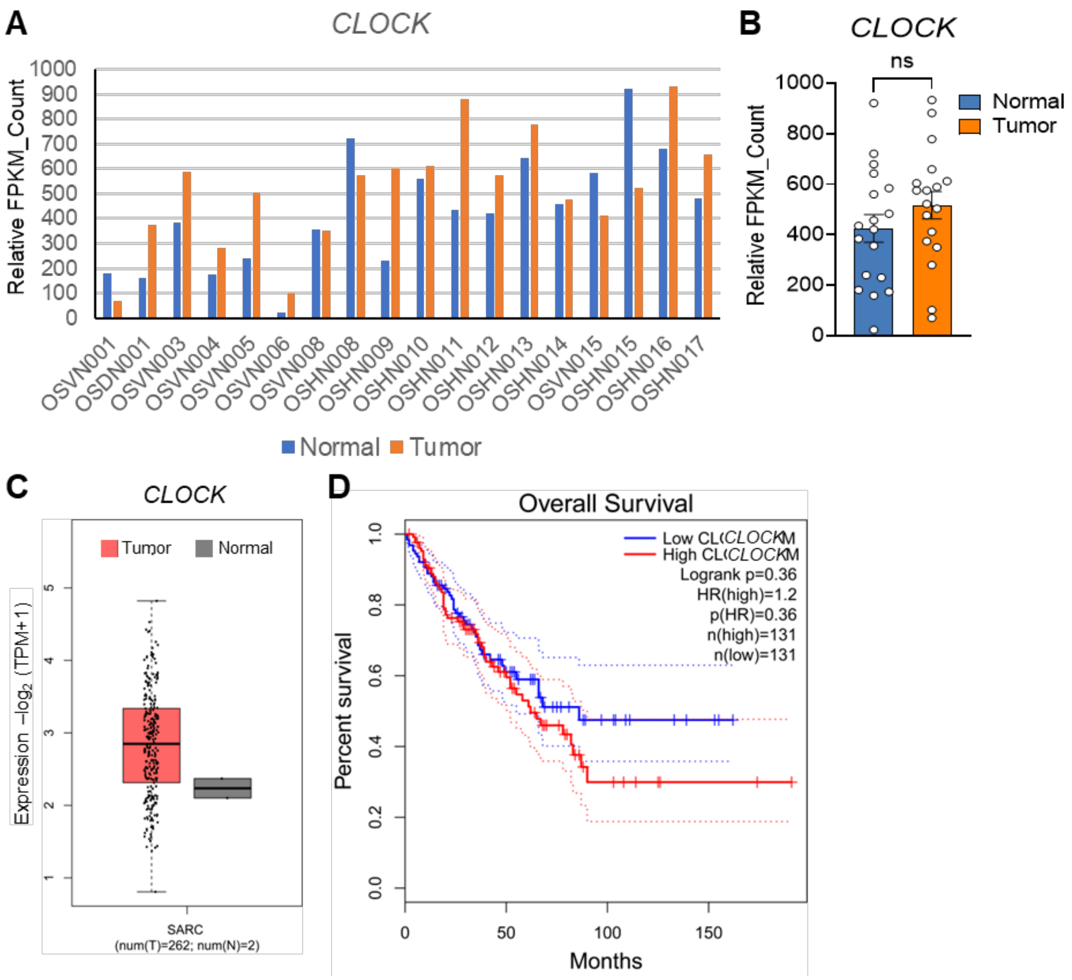

Figure S9. **Differential expressions of *CLOCK* in normal and tumor tissues from human OS patients.** (**A**) Publicly available RNA sequencing data (GSE99671) was used to generate expression profiles for *CLOCK* in normal (blue) and tumor (orange) tissues harvested from bone samples of 18 individual osteosarcoma (OS) patients. FPKM: Fragments Per Kilobase of Transcript per Million mapped reads. (**B**) Bar graph quantifying the expressions of *CLOCK* in the normal and tumor tissues shown in (A). (**C**) Box plot analysis of the expressions of *CLOCK* in tumor (T, crimson) and normal (N, dark gray) tissues from human sarcoma (SARC) patients. Plots were generated with data from Gene Expression Profiling Interactive Analysis (GEPIA; http://gepia.cancer-pku.cn/). Statistical significance was determined using one-way ANOVA with Tukey's multiple comparisons test. ns: not significant. (**D**) Survival curve for SARC patients based on the expression levels of CLOCK was generated using GEPIA. Analysis of the survival data was performed with the Cox proportional hazard model, and Kaplan–Meier (KM) curve parameters were applied to evaluate the significance of gene expression on patient outcomes. KM plot includes the hazard ratio (HR), Cox model p-value, and logrank p-value. TPM, Transcripts Per Million.

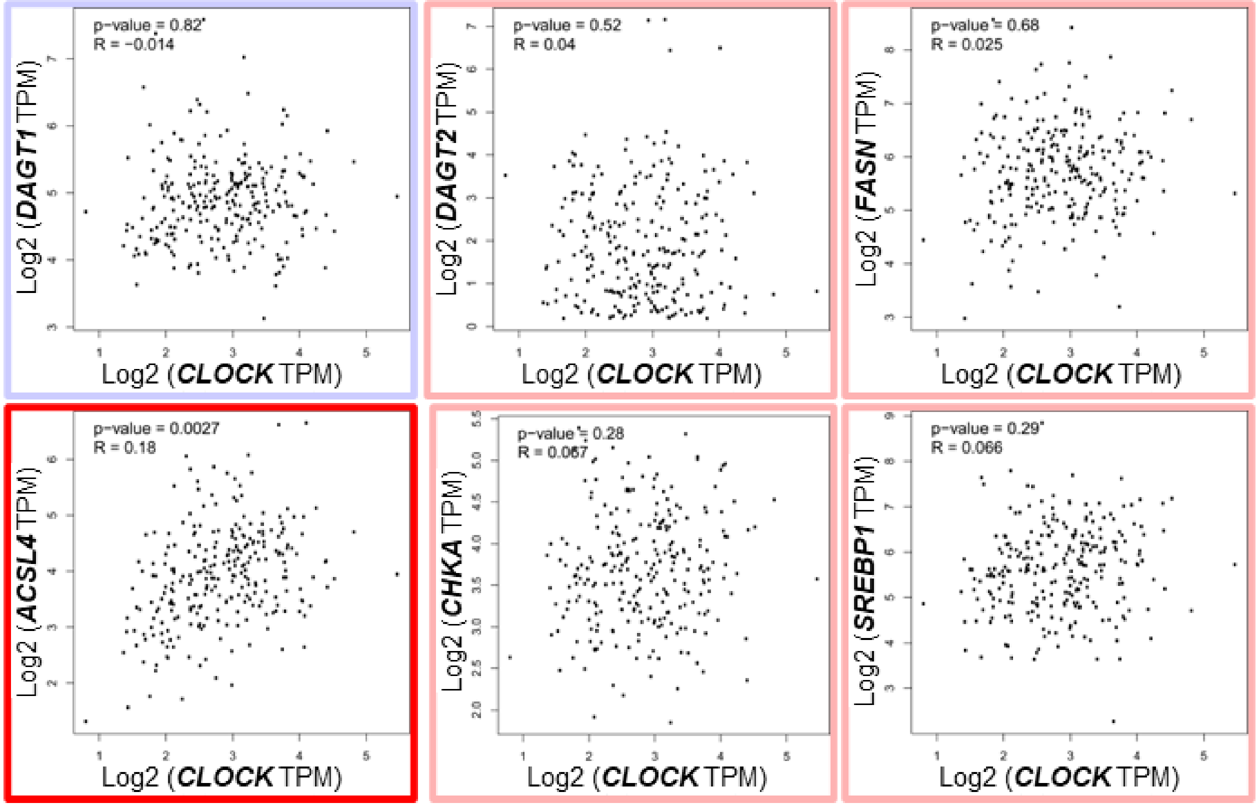

Figure S10. Correlation of CLOCK with lipid droplet biogenesis-associated gene expression in human OS patients. Correlations between the expressions of *CLOCK* and genes associated with lipid droplet (LD) biogenesis (*DGAT1*, *DGAT2*, *FASN*, *ACSL4*, *CHKA*, *SREBP1*) in human osteosarcoma patient samples were determined. Data were sourced from the Gene Expression Profiling Interactive Analysis (GEPIA; http://gepia.cancer-pku.cn/). Genes with positive and negative correlations, based on purity-adjusted partial Spearman's rho values, are highlighted with light red and light blue boxes, respectively. Genes with significantly positive correlations ($p < 0.05$) are marked with bold red boxes. TPM, Transcripts Per Million.
